# A multi-modal deep learning model for prediction of Ki-67 for meningiomas using pretreatment MR images

**DOI:** 10.1038/s41698-025-00811-1

**Published:** 2025-01-21

**Authors:** Chaoyue Chen, Yanjie Zhao, Linrui Cai, Haoze Jiang, Yuen Teng, Yang Zhang, Shuangyi Zhang, Junkai Zheng, Fumin Zhao, Zhouyang Huang, Xiaolong Xu, Xin Zan, Jianfeng Xu, Lei Zhang, Jianguo Xu

**Affiliations:** 1 Department of Neurosurgery, West China Hospital, Sichuan University, West China Hosptial, No. 37, GuoXue Alley, Chengdu, China; 2 Diseases of Women and Children, Sichuan University, Ministry of Education, No. 20, section 3, Renmin South Road, Wuhou District, Chengdu, China; 3 Department of Radiology, West China Second University Hospital, Sichuan University, No. 20, section 3, Renmin South Road, Wuhou District, Chengdu, China; 4 College of Computer Science, Sichuan University, Chengdu, China; 5 Department of Neurosurgery, Third People’s Hospital of Mianyang/Sichuan Mental Health Center, No. 190, East Section of Jiannan Road, Mianyang, China

**Keywords:** Medical research, Oncology

## Abstract

This study developed and validated a deep learning network using baseline magnetic resonance imaging (MRI) to predict Ki-67 status in meningioma patients. A total of 1239 patients were retrospectively recruited from three hospitals between January 2010 and December 2023, forming training, internal validation, and two external validation cohorts. A representation learning framework was utilized for modeling, and performance was assessed against existing methods. Furthermore, Kaplan–Meier survival analysis was conducted to investigate whether the model could be used for tumor growth prediction. The model achieved superior results, with areas under the curve (AUCs) of 0.797 for internal testing and 0.808 for generalization, alongside 0.756 and 0.727 for 3- and 5-year tumor growth predictions, respectively. The prediction was significantly associated with the growth of asymptomatic small meningiomas. Overall, the model provides an effective tool for early prediction of Ki-67 and tumor volume growth, aiding in individualized patient management.

## Introduction

Meningiomas are the most common primary intracranial tumors with an annual incidence of 9.73 per 100,000 individuals^1,2^. Magnetic resonance imaging (MRI) is the cornerstone in patient management as it plays the most important role in tumor diagnosis, follow-up, and treatment planning^3^. For symptomatic meningioma, complete surgical resection with/without adjuvant radiotherapy is recommended as it can reach favorable prognosis in most cases^4^. In contrast, asymptomatic meningiomas of small size (maximal diameter less than 3 cm) are conservatively managed through radiological follow-up, with the decision for active intervention determined by development of clinical symptoms or radiological evidence of tumor growth^5^. Recognizing the importance in both prognostic risk stratification and therapeutic decision making, there is a growing emphasis on achieving individualized patient management and prognosis evaluation^6^.

Recent studies have provided accumulated evidence regarding the clinical significance of Ki-67 index in individualized patient care. As a classical bio-marker of cell proliferation, it can be used for identifying asymptomatic small meningioma patients who need early intervention. Additionally, it holds promise as prognostic indicator related to both increased recurrent risk after surgical resection^7^ and poor progression-free survival (PFS) after post-operative stereotactic radiosurgery^8^. The gold standard for Ki-67 assessment remains immunohistochemistry staining of tumor specimens obtained from biopsy or surgery^9^. Nevertheless, the invasiveness of these approaches imposes significantly increased physical burden and risks of complication. Hence, there is a critical need to identify an alternative approach for accurately assessing Ki-67 expression in meningioma cases based on information obtained from MRI.

Previous research has identified a series of radiological characteristics on routine MRI that independently correlated with Ki-67 expression^10^. Parameters obtained from advanced MR technology, such as the relative apparent diffusion coefficient (rADC), may also potentially serve as independent predictors^11–14^. Yet, accurate prediction by human being remains challenging as these indicators remain controversial, imprecise, relying on clinical expertize^15^, nor advanced MR technology is routinely performed in patient management.

Multi-modal representation learning models have drawn great attention in recent years. Presented as Transformer-based structure^16,17^, they have demonstrated a remarkable adaptability in medical-related tasks and offered distinctive advantages. By leveraging cross-attention module to interact and integrate features of multi-modal inputs, both performance and generalization can be enhanced with redundancy of information, therefore representing the logical next step^18–20^. Returning to this topic, radiological characteristics, radiomics features, and deep learning features are interconnected but distinct from the perspective of medical imaging analysis^21^. Nevertheless, to the best of knowledge, the potential of multi-modal representation learning in prediction of Ki-67 on MRI for meningiomas has not been investigated yet.

Thus, in this study, with a large multicenter dataset collected from three healthcare institutions, we aimed to developed and validated a multi-modal representation learning model to predict the Ki-67 index for meningiomas.

## Results

### Resected meningioma cohort

Table 1 provides a full overview of the patient demographics and baseline clinical characteristics. Generally, a total of 1008 patients with resected meningioma were selected in this study. The mean patient age was 55.2 year (range: 27–76), and 70.0% (*n* = 705) of the study cohort was female. A total of 296 patients presented with skull base meningioma, and the other 712 patients presented with non-skull base meningioma.

**Table 1:** Baseline demographic and clinical characteristics of the included patients

	Dataset A	Dataset B	Dataset C
Total	723	285	231
Ki-67			
≥5% (high group)	167 (23.1%)	50 (17.5%)	-
<5% (low group)	556 (76.9%)	235 (82.5%)	-
Sex			
Female	502 (69.4%)	203 (71.2%)	71 (30.7%)
Male	221 (30.6%)	82 (28.8%)	160 (69.3%)
Age	56.0 (Range: 32–76)	53.1 (Range: 27–72)	43.9 (Range: 27–83)
Tumor volume (cm^3^)	36.6 (Range: 4.7–337.2)	32.2 (Range: 3.1–129.4)	3.2 (Range: 0.15–9.73)
Location			
Cerebral convexity	406 (56.2%)	141 (49.5%)	85 (36.8%)
Falx	118 (16.3%)	47 (16.5%)	43 (18.6%)
Skull base	199 (27.5%)	97 (34.0%)	103 (44.6%)
Laterality			
Left	308 (42.6%)	125 (43.9%)	97 (42.0%)
Right	327 (45.2%)	136 (47.7%)	110 (47.6%)
Midline	88 (12.2%)	24 (8.4%)	24 (10.4%)
WHO grade			
WHO I	540 (74.7%)	243 (85.3%)	-
WHO II	156 (21.6%)	36 (12.6%)	-
WHO III	27 (3.7%)	6 (2.1%)	
Peritumoral edema			
EI = 1	372 (51.5%)	149 (52.3%)	209 (90.5%)
1 < EI < 2	172 (23.7%)	52 (18.2%)	9 (3.8%)
2 ≤ EI < 3	100 (13.8%)	53 (18.6%)	7 (3.0%)
EI ≥ 3	79 (10.9%)	31 (10.9%)	6 (1.7%)
CSF cleft surrounding tumor	496 (68.6%)	207 (72.6%)	202 (87.4%)
Absent capsular enhancement	179 (24.8%)	29 (10.2%)	22 (9.5%)
Intra-tumoral necrosis	173 (23.9%)	60 (21.1%)	12 (5.2%)

*EI* edema index, *CSF* cerebral spinal fluid.

There were 791 patients with Ki-67 < 5% and 217 patients with Ki-67 of ≥ 5%. The univariate analysis of the relationship between radiological characteristics and Ki-67 index is provided in the Supplementary Table 3. As suggested, meningiomas with Ki-67 ≥ 5% were larger in volume compared to tumors with Ki-67 < 5% (mean 46.3 ± 48.2 and 27.0 ± 25.9 cm^3^; *p* < 0.001), Similarly, meningiomas with Ki-67 ≥ 5% had significantly larger EI compared to tumors with Ki-67 < 5% (*p* < 0.001), and also more likely to present intra-tumoral necrosis (*p* = 0.008). Whereas, there was no statistic difference regarding to CSF cleft surrounding tumor (*p* = 0.493) or absent capsular enhancement (*p* = 0.315) between two groups. As presented in the Supplementary Table 4, multivariate analysis suggested that intra-tumoral necrosis was the only characteristic independently associated with Ki-67 expression (Odds ratio (OR) = 4.048; 95CI: 2.878–5.694; *p* < 0.001).

### Model performance and generalization

**Fig. 1:**
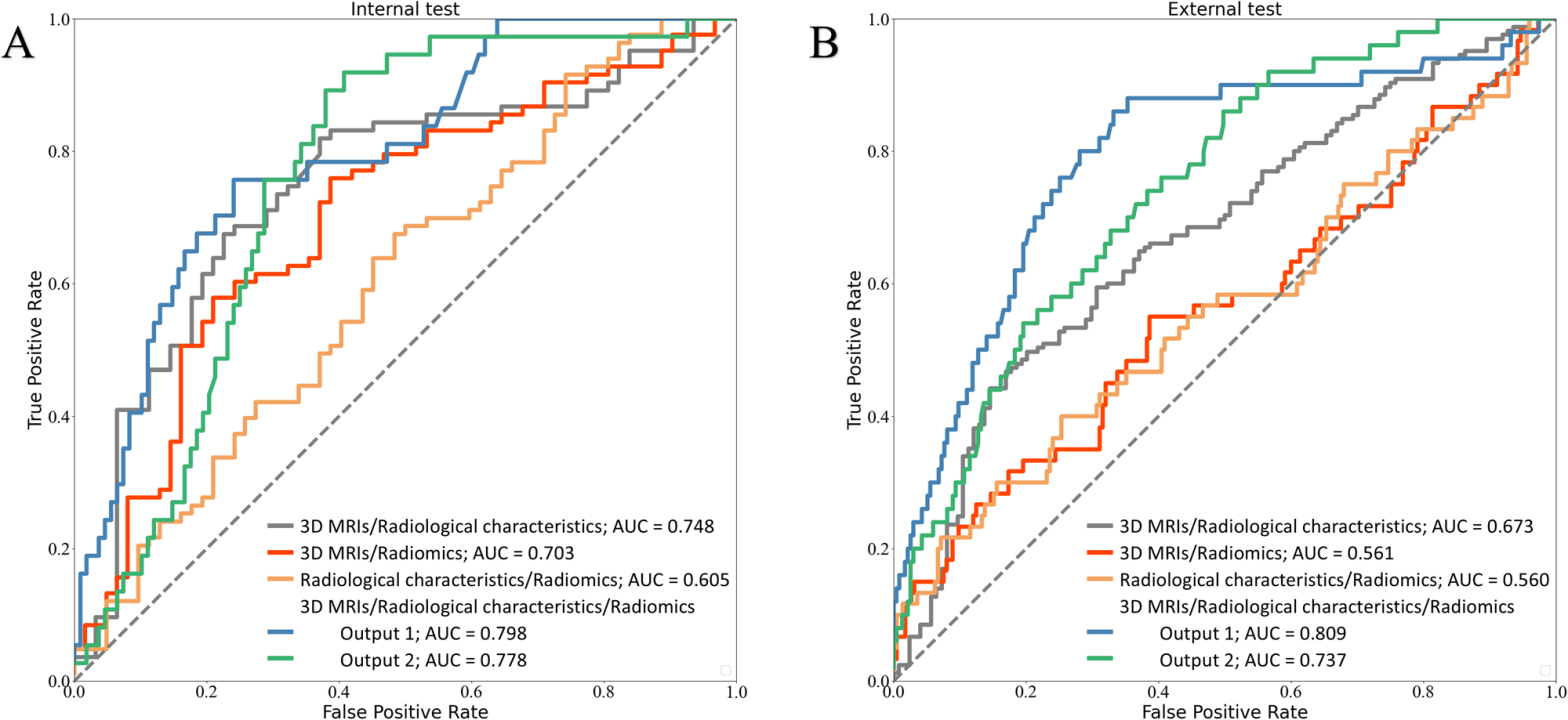
Performance analysis of various models in predicting Ki-67 index. Receiver operating characteristics curves of different combinations of modalities as input with closest performance to the average performance of all 50 models in the internal validation cohort (**A**) and external validation cohort (**B**).

The results of ablation experiment to determine the suitable input are provided in the Supplementary Note 3. Table 2 presents the results of setting different combinations of modalities as input. The results suggested that setting combination of 3D MRIs, radiological characteristics, and Radiomics as input was the best one as it achieved the highest AUC as well as the MCC. More specifically, Output 1, utilizing MRI and radiological characteristics as input, exhibited relatively better performance. In the internal test, the model achieved an AUC of 0.797 (95CI: 0.758–0.837), accuracy of 0.748, sensitivity of 0.739, specificity of 0.760, F1 score of 0.746, and MCC of 0.495. The metrics demonstrated robustness in the external test as well, with an AUC of 0.808 (95CI: 0.726–0.895), accuracy of 0.769, sensitivity of 0.756, specificity of 0.771, F1 score of 0.755 and MCC of 0.537. The ROC curves of the model with closest performance to the average performance are illustrated in Fig. 1.

**Table 2:** A summary of performances of the predictive model in classifying high-Ki-67 meningiomas from low in internal test as well as external test

Inputs	Dataset	AUC (95CI)	Accuracy (95CI)	Sensitivity (95CI)	Specificity (95CI)	F1 score (95CI)	MCC (95CI)
3D MRIs and radiological characteristics							
	Internal	0.749	0.712	0.687	0.747	0.732	0.431
		(0.706–0.795)	(0.674–0.75)	(0.639–0.735)	(0.689–0.806)	(0.691–0.772)	(0.352–0.513)
	External	0.672	0.639	0.600	0.692	0.590	0.271
		(0.633–0.71)	(0.606–0.677)	(0.561–0.649)	(0.643–0.738)	(0.559–0.627)	(0.210–0.348)
3D MRIs and radiomics							
	Internal	0.701	0.679	0.729	0.611	0.751	0.324
		(0.652–0.737)	(0.632–0.715)	(0.675–0.771)	(0.557–0.672)	(0.705–0.792)	(0.237–0.401)
	External	0.544	0.617	0.501	0.641	0.598	0.136
		(0.480–0.604)	(0.586–0.646)	(0.412–0.588)	(0.607–0.671)	(0.549–0.662)	(0.066–0.216)
Radiological characteristics and radiomics							
	Internal	0.589	0.431	0.651	0.385	0.502	0.027
		(0.533–0.645)	(0.395–0.479)	(0.56–0.76)	(0.336–0.437)	(0.461–0.553)	(−0.055–0.107)
	External	0.580	0.599	0.459	0.628	0.523	0.134
		(0.472–0.601)	(0.556–0.636)	(0.353–0.529)	(0.585–0.671)	(0.477–0.611)	(0.055–0.218)
3D MRIs, radiological characteristics, and radiomics							
Output 1	Internal	0.797	0.748	0.739	0.760	0.746	0.495
		(0.758–0.837)	(0.715–0.792)	(0.687–0.795)	(0.705–0.803)	(0.708–0.787)	(0.417–0.571)
	External	0.808	0.769	0.756	0.771	0.755	0.537
		(0.726–0.895)	(0.733–0.811)	(0.625–0.875)	(0.730–0.816)	(0.695–0.828)	(0.437–0.644)
Output 2	Internal	0.776	0.678	0.701	0.649	0.738	0.356
		(0.735–0.815)	(0.638–0.722)	(0.639–0.765)	(0.590–0.707)	(0.695–0.778)	(0.280–0.432)
	External	0.736	0.620	0.752	0.598	0.751	0.280
		(0.654-0.824)	(0.561–0.667)	(0.624–0.874)	(0.551–0.653)	(0.687–0.812)	(0.179–0.382)

*AUC* area under the curve, 95*CI* 95% confidence interval, *MCC* Matthews correlation coefficient.

Supplementary Figure 2 presents representative Grad-CAM and cross-attention maps for predictions of both high Ki-67 and low Ki-67 cases. As suggested, the Grad-CAM analysis indicated that the MRI block highlighted the tumor region and extracted high-level features for prediction. Similarly, the cross-attention map demonstrated effective interaction between the blocks, wherein the model selectively attended to related MRI regions consistent with radiological characteristics during output generation. The weights of combined loss function in this sequential task were W _mri_ = 0.5, W _radiological characteristics_ = 0.4, W _radiomics_ = 0.1, respectively.

### Comparison with clinical model and the existing methods

The clinical model built with logistic regression presented performance with AUC of 0.664 (95CI: 0.624–0.710) in the internal test, and 0.506 (95CI: 0.439–0.587) in the external test. As comparing to the previously reported deep learning or machine learning methods, the best machine learning model achieved AUC of 0.746 (95CI: 0.717–0.780), accuracy of 0.655, sensitivity of 0.675, specificity of 0.629, F1 score of 0.675, and MCC of 0.299 in internal test, and AUC of 0.676 (95CI: 0.585–0.769), accuracy of 0.817, sensitivity of 0.314, specificity of 0.915 F1 score of 0.423, and MCC of 0.351 in external test. More detailed results were presented in the Supplementary Table 5 and Supplementary Table 6.

### Prognostic value in tumor growth prediction

**Fig. 2:**
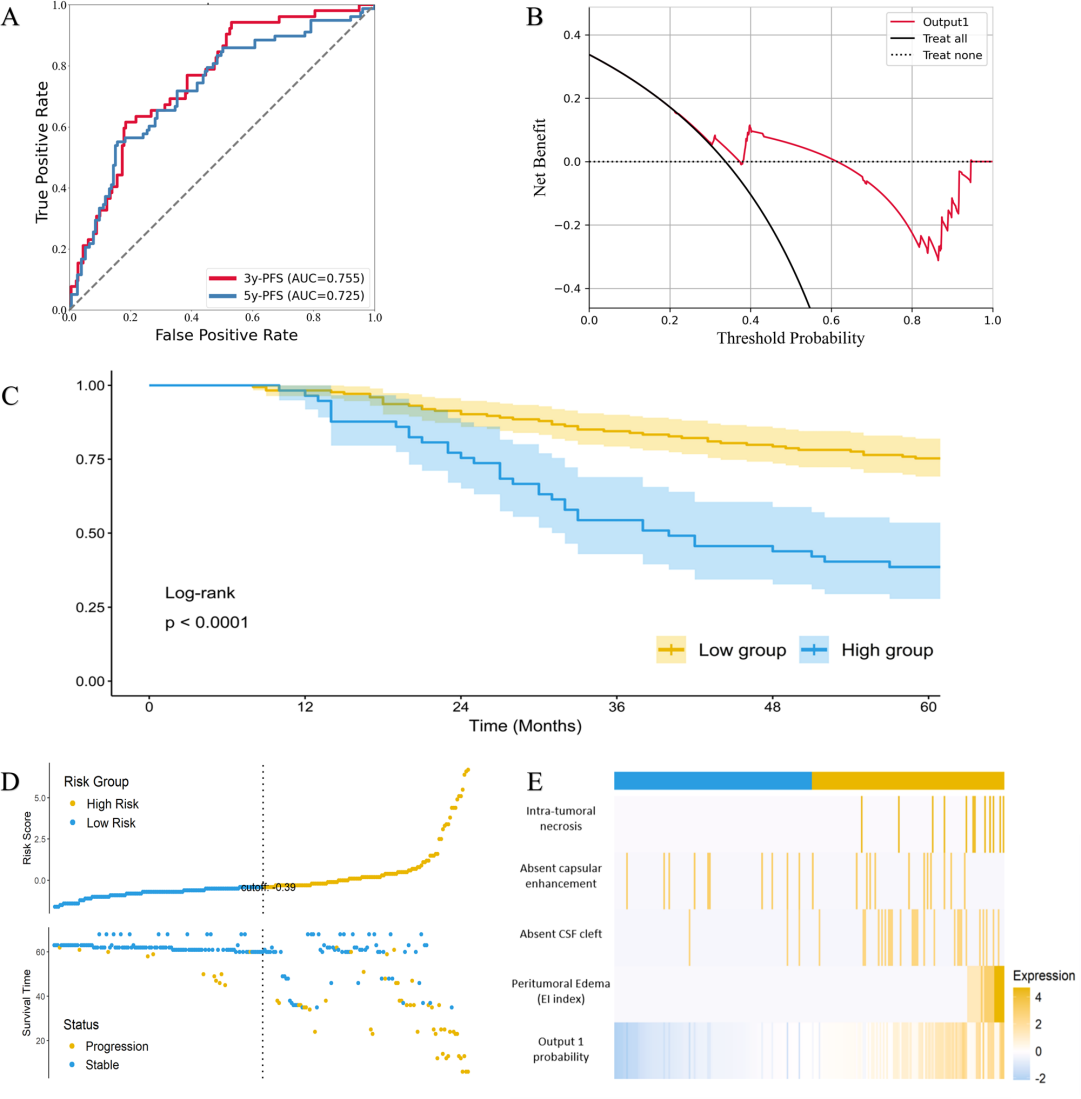
Correlation of the predicting probability with tumor volume growth after radiological diagnosis of meningiomas. **A** ROC curves of the model in 3-years tumor growth prediction and 5-years tumor growth prediction; **B** Decision curves of Output 1 in predicting tumor growth of asymptomatic small meningiomas after radiological diagnosis in 5 years; **C** K–M curves of the low-risk group and high-risk group stratified by model; **D** the corresponding tumor growth status and time for each patient stratified by output 1; **E** Heatmaps of predictors according to multivariate Cox regression analyzes between high-risk and low-risk groups.

Significant tumor volume growth was observed in 22.5% (52 cases) within three years, and 33.8% (78 cases) within five years. Generally, as shown in Table 3, the predictive model (output 1) exhibited promising potential in anticipating tumor growth, achieving the following metrics for three-year tumor growth prediction (Fig. 2a): AUC of 0.756 (95CI: 0.722–0.793), accuracy of 0.753, sensitivity of 0.503, specificity of 0.825, F1 score of 0.641, and MCC of 0.321. For five-year tumor growth prediction, the model yielded an AUC of 0.727 (95CI: 0.699–0.756), accuracy of 0.721, sensitivity of 0.452, specificity of 0.859, F1 score of 0.588, and MCC of 0.305.

**Table 3:** A summary of performances of the developed model in predicting tumor growth in 3 year and 5 year

Inputs	Dataset	AUC (95CI)	Accuracy (95CI)	Sensitivity (95CI)	Specificity (95CI)	F1 score (95CI)	MCC (95CI)
Overall							
	In 3 years	0.756	0.753	0.503	0.825	0.641	0.321
		(0.722–0.793)	(0.727–0.779)	(0.442–0.577)	(0.804–0.855)	(0.586–0.702)	(0.302–0.439)
	In 5 years	0.727	0.721	0.452	0.859	0.588	0.305
		(0.699–0.756)	(0.697–0.745)	(0.396–0.513)	(0.830–0.882)	(0.547–0.633)	(0.247–0.364)
Skull base meningioma							
	In 3 years	0.786	0.827	0.339	0.889	0.465	0.317
		(0.749–0.828)	(0.804–0.848)	(0.231–0.423)	(0.868–0.912)	(0.352–0.563)	(0.231–0.425)
	In 5 years	0.687	0.717	0.259	0.909	0.369	0.099
		(0.651–0.728)	(0.717–0.756)	(0.217–0.303)	(0.889–0.929)	(0.300–0.422)	(0.027–0.165)
None skull base meningioma							
	In 3 years	0.717	0.696	0.551	0.762	0.628	0.297
		(0.682–0.753)	(0.670–0.722)	(0.503–0.597)	(0.734–0.785)	(0.594–0.667)	(0.239–0.358)
	In 5 years	0.742	0.726	0.545	0.803	0.632	0.356
		(0.71–0.777)	(0.7–0.755)	(0.496–0.588)	(0.768–0.833)	(0.597–0.667)	(0.298–0.415)

*AUC* area under the curve, 95*CI* 95% confidence interval, *MCC* Matthews correlation coefficient.

In survival analysis, the decision curves illustrated that the developed model was the more useful prediction model for clinical decision-making (Fig. 2b). Kaplan-Meier survival curves and log-rank analysis also demonstrated a significant statistical difference (*p* < 0.001) between the two groups categorized by the model (Fig. 2c). Subgroup analysis also suggested that our model can achieve satisfactory patient stratification, whether for skull-base meningiomas (*p* = 0.015) or non-skull-base meningiomas (*p* < 0.001) (Table 3 and Supplementary Figure 3). Furthermore, as presented in the Fig. 2d, the corresponding predicting probability along with their survival status and survival time for each patient in Dataset 3 is visualized. Supplementary Table 7 and Fig. 2e provided the results of univariate and multivariate Cox regression analyzes of predictors of tumor volume growth, suggesting that the prediction could be considered as an independent predictor (HR = 1.996; 95CI: 1.117–3.568; *p* = 0.020).

## Discussion

This large-scale multicenter study proposed a multi-modal representation learning model to pre-surgically predict the Ki-67 index of meningiomas on MRI. The model achieved good performance along with generalization, and could be regarded as a useful tool to facilitate individualized management of asymptomatic meningioma patients.

Medical image analysis technology aims at capturing complicated patterns of tumor images that are imperceptible to naked eye^22–24^, previous studies applied voxel-level analysis and artificial intelligence (AI) algorithms for modeling. Both radiomic-based machine learning^10,25,26^ and deep learning^27^ approaches demonstrated the feasibility with their results, suggesting a non-invasive method of predicting the Ki-67 index in meningioma patients, as outlined in Supplementary Table 8. However, limited feasibility is still the primary concern from the perspective of clinical translation. It can be presented from three aspects. The first one is the sharp decline in model performance metrics when transitioning well-trained model from internal validation to independent external test. The objectives in medical images, especially referred to MRI, can be dramatically various in image pattern due to heterogeneous acquisition protocols, different institutions, or distinctive patient populations^28,29^. These domain shifts challenge model performance but must be overcome in clinical translation. The second one is the incapability in handling missing data. The standard protocols for radiomics-related research have not yet achieved consensus, and features selected for modeling were considerably variate. Moreover, the complicated but highly variant procedures of image pre-processing are also common and significantly lead to low reproducibility of feature extraction. Whereas, the real clinical landscape presents a far more complicated scenario. Given the highly fixed nature of the model’s input, the absence of any single feature or modality, a situation highly likely to be encountered in clinical settings, will render the model unusable^28^. The third one is that no study has demonstrated how can the model facilitate clinical decision-making yet. Although there is a consensus that predicting Ki-67 is of great clinical significance, it’s still unclear for clinicians that if they can make therapeutic decision consistent with the latest guidelines when using AI model. Therefore, our results can be interpreted as important as it provided critical information from following distinctive aspects.

The first one is that the developed model could be seamlessly integrated into workstations that commonly used in the healthcare institutions, and supported an automated, accurate, and stable clinical management workflow. It should be noted that the advantage of our model in flexibly accommodating various data scenarios. The combination of radiomics with machine learning is the most common method used in previous studies. However, these handicraft features necessitate delineating tumor segmentation, which is subjective, time-consuming, low-reproducing, and laborious. In contrast, MRI and radiological characteristics are much more easily obtained in routine diagnostic workflow. Compared with previous studies, the inputs of our model were more flexible as they were fed into the network separately rather than simultaneously, and the dropout modules and the auxiliary output module were introduced to obtain the intermediate outputs. So, the clinicians were able to obtain the prediction with/without radiomics input, and it is rational to say that our model is more adaptable to the practical use of clinicians.

The second perspective is that it provides valuable insights into a novel method of handling multi-modal data to the field of CAD research. Low generalization is a major concern of previous studies. For instance, a deep learning model might thrillingly achieve an AUC of 0.966 in internal test, while its performance would notably deteriorate to an AUC of 0.591 in external test^27^. Empirically, multi-modal models are anticipated to address this question owing to potential informative redundancy between modalities. Previous studies also indicated the superiority of clinical-radiomic models over single radiomic models^10,25^. However, directly setting multi-modal information as input is not mathematically rational, as it cannot capture complex relationships between the different modalities. Besides, it may technically lead to challenge in handling high-dimensional feature spaces which results in computational inefficiency, increased training time, and overfitting^30^. Therefore, we hypothesized that the multi-modal representation learning with cross-attention was a viable solution. More specifically, the design of our model was to use the radiological characteristics to guide the attention of deep learning block to the related regions of the MRI, and the deep learning block could consequently extract more enriched and informative representations of the input data. So, the predictions of the model were made by integrating information from multiple modalities into a cohesive representation. The results suggested the improvement of multi-modal learning in superior performance comparing to the existing methods, and indicated a new method to use multi-modal inputs to enhance both reproducibility and performance of deep learning model.

Thirdly, this research also provided concrete evidence showing the potential of AI model in aiding clinical decision-making. Regarding the prediction of Ki-67 expression in meningiomas, one of the most important clinical relevance is its application in predicting tumor growth of asymptomatic meningiomas. As reported by previous studies, the therapeutic decision of asymptomatic tumor was commonly determined by the location, as it was rather difficult for neurosurgeons to identify the easily growing one^15^. Comparatively, the results of our model suggested to be thrilling with AUC of 0.756 and 0.727 in prediction, as well as significant statistical difference in PFS between the stratified groups. Also, within these results, two key points warrant attention: 1) Follow-up outcomes indicated that the majority of patients’ tumors did not exhibit significant volume growth (three years: 77.5%; five years: 66.2%); 2) Similar to previous studies, although the model maintained rather balanced performance metrics during training, specificity was notably higher than sensitivity in the external test. These findings demonstrated that when using the model to make therapeutic decision for asymptomatic meningiomas, the decisions might lean towards conservatism as it encouraged clinicians to opt for follow-up observation rather than active intervention. To be more specific, for patients predicted to be at high risk, intervention should be performed actively as the high specificity; whereas, for patients predicted to be at low risk, regular follow-up should still be maintained to monitor tumor progression as the rather high false negative rate. It seems clinically rational as the therapeutical decisions based on this model aligns with the diagnostic and treatment recommendations outlined in the latest guidelines^2,5^. Whereas, further research is still warranted to explore methods for enhancing the model’s sensitivity in identifying such patients.

There is another contradicted point should be also worthy noted. As evidenced by the results, including radiomics in network training does indeed improve model performance, but during the inference phase, it negatively impacts the model’s decision-making. The most common reason for this is that this modality provides information redundancy and stabilizes the model optimization. But due to the redundancy or even noise it carries, removing it in inference may lead the model to focus more on other more valuable modalities, thereby improving performance. These findings suggested that the radiomics features might be treated as noise rather than useful features in this model, and it acted more like an implicit regularization effect rather than useful inputs. This could be explained from following perspectives: firstly, the CNN module is equally adept at extracting local features (such as texture and shape), partially replacing the informative role of radiomics; secondly, as the tabular data, radiomics are known for its high dimensionality, heterogeneity, and correlative relationships, but the neural networks often struggle to handle them effectively^31^. Therefore, considering both the model’s performance and clinical requirements, we concluded that output 1 was the most suitable one.

Our study possesses several limitations. Firstly, as retrospective research, the selection bias was inherent and unavoidable. Secondly, limited by the image quality of the dataset, we only included T1C images of MP-RAGE in this modeling. Future studies should investigate the method of integrating multi-sequences and advanced MR imaging to enhance the model’s performance. Thirdly, there is still deficiency from the perspective of clinical validation. Studies have shown that Ki-67 also holds significance in predicting patient prognosis (such as tumor recurrence after surgical resection and/or gamma knife treatment). Therefore, more research is required to investigate how the model can facilitate therapeutic decision-making for surgically resected meningioma patients in the future. Fourth, while Ki-67 is a valuable biomarker, WHO grade is also a well-established, clinically actionable parameter that significantly predicts recurrence and outcome. We do not include this parameter in our model because there have already been numerous studies that had successfully predicted WHO grades with promising results. Moreover, Transformer-based models are typically applied to large language datasets. Although our training set contains 578 patients, high-grade meningiomas account for only 25.3%, leading to significant imbalanced data distribution. Therefore, the comparison between two biomarkers in terms of prognostic value is still absent, and future study is still required to investigate which one is better for meningioma patients.

## Methods

### Study overview

**Fig. 3:**
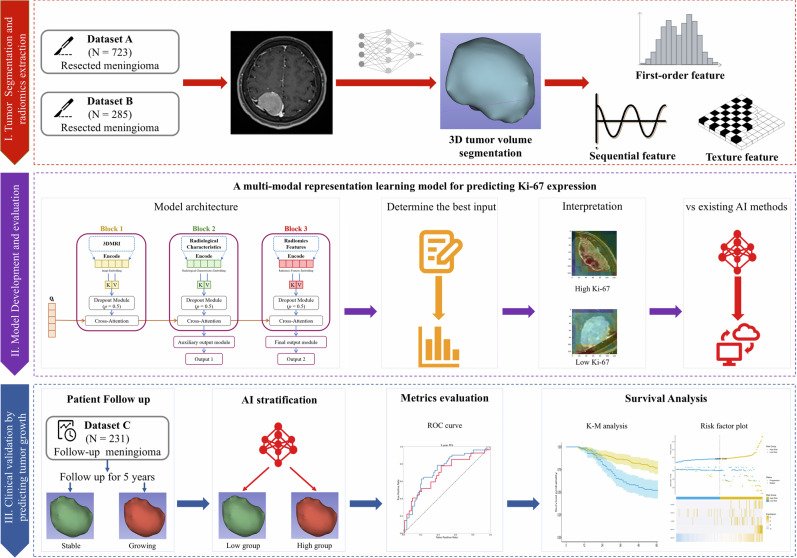
The flowchart of model training, assessment, and clinical validation.

Figure 3 illustrates the overview of the current research. Briefly, this study consisted of two steps: first, we collected surgically resected meningioma patients from two institutions to develop the multi-modal representation learning model, and evaluated its performance in both internal set and external set; then, to further demonstrate that how exactly our model could be used to aid in the clinical work, we collected a follow-up asymptomatic small meningioma from the third institution, and investigated that if the stratification by the developed model could show significant difference in patient survival.

### Input definition

**Fig. 4:**
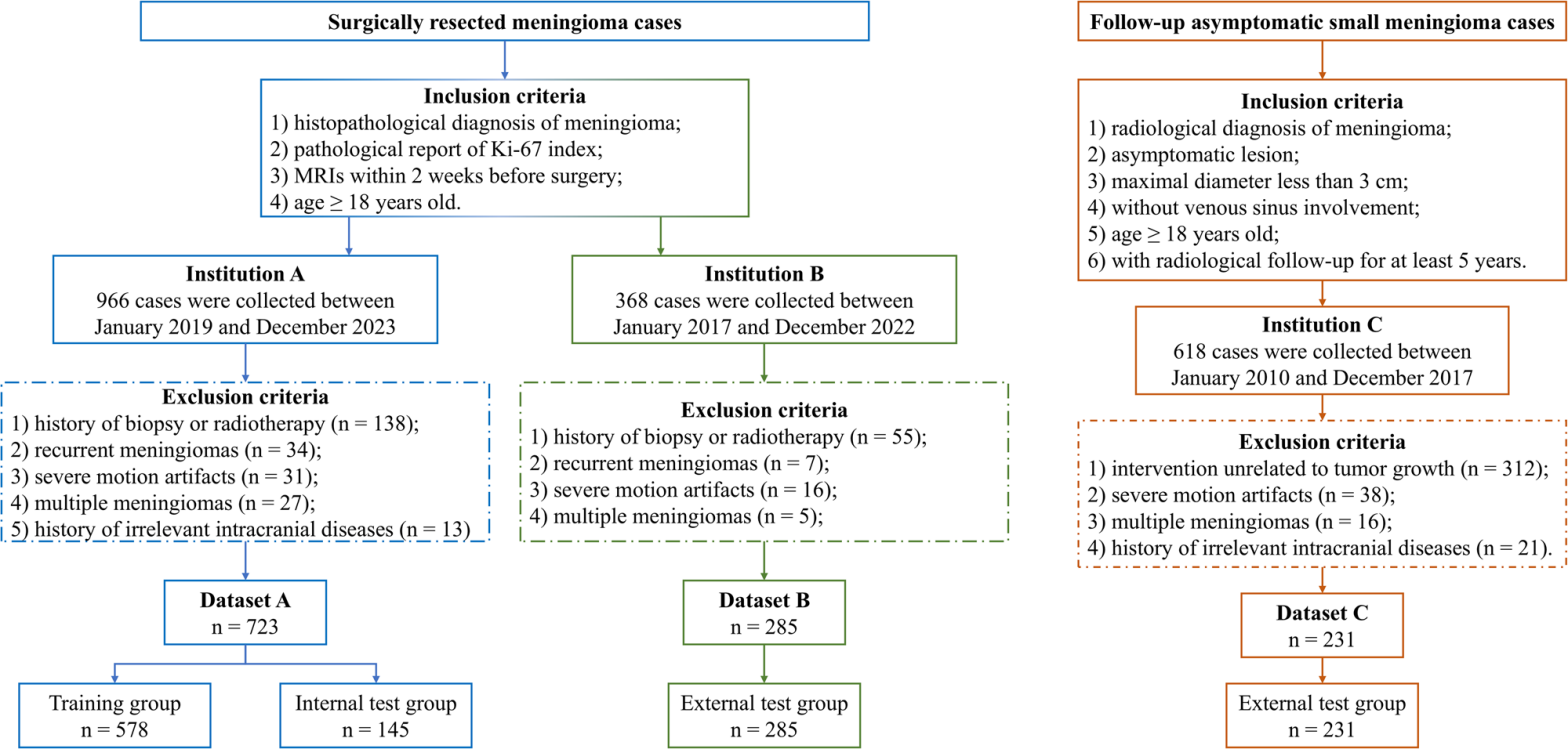
Patient recruitment workflow of this research.

Patients who underwent surgical resection for meningioma were recruited from West China Hospital *Dataset A)* and the Third People’s Hospital of Mianyang *(Dataset B)* between January 2017 and December 2023. The inclusion criteria were set as follows: (1) histopathological diagnosis of meningioma according to the 2021 WHO classification; (2) immunohistochemical record of Ki-67 index; (3) standardized MR scans within 2 weeks before surgery; (4) age ≥ 18 years old. The exclusion criteria included: (1) history of biopsy or radiotherapy; (2) recurrent meningiomas; (3) severe motion artifacts; (4) multiple meningiomas; (5) history of other intracranial diseases, such as subarachnoid hemorrhage. Based on above criteria, a total of 723 cases were identified from Center A, and 285 cases were identified from Center B, respectively. Ki-67 index was assessed by immunohistochemistry using an avidin-biotin-peroxidase complex method by using Aperio IHC image analysis software, and patients were stratified by a cutoff of 5%, a threshold deemed clinically relevant and used in most of previous studies^26,32,33^. The patient recruitment workflow of this research is shown in Fig. 4.

Four MR sequences were acquired and exported from Picture Archive and Communication System (PACS), including T1-weighted imaging (T1WI), T2-weighted imaging (T2WI), fluid attenuated inversion recovery (Flair) and contrast-enhanced T1-weighted imaging (T1C). The scanning protocols of each dataset are provided in the Supplementary Note 1, which demonstrates that the MRIs were acquired from scanners of different vendors along with a wide variety of acquisition settings.

There is the noticeable difference in slice thickness between T1WI, T2WI, and FLAIR (5 mm) and T1C (1 mm), which means that the information they contain is quite limited. Therefore, quantitative image analysis is not suitable for these sequences and only T1C was set as input. Image preprocessing for each of the patients involved voxel resampling to 1 × 1 × 1 mm^3^, and N4 bias field correction for intensity nonuniformity adjustment. The intensity values for all image volumes were scaled in the range [0 255].

The evaluation of radiological characteristics was performed by two senior neuro-radiologists from two institutions (with more than 15 years’ experience in image reading) on pre-treatment MRI. The results were also independently reviewed by a neuro-radiologist with 20 years of experience from another Hospital. It should be noted that some radiological characteristics, like signal intensity and tumor size, can be quantified as radiomics features. Therefore, we focused on four “yes or no” characteristics and set them as inputs, including peritumoral edema (presenting as Edema Index, EI), intra-tumor necrosis, cerebral spinal fluid (CSF) cleft surrounding tumor, and capsular enhancement (Supplementary Fig. 1).

As the same reason described in the 2.2.1, T1WI, T2WI, and FLAIR images were excluded in quantitative radiomics extraction. Here, tumor segmentation was performed on T1C images using an automatic deep learning approach that the authors developed previously^34^. Afterwards, two senior neuro-radiologists with 15 and 20 years of experience in image reading, blinded to clinicopathological information, manually adjusted the preliminary segmentation using 3D Slicer software, and any discrepancies were resolved through consensus. Finally, a total number of 1218 radiomics features were retrieved from tumor segmentation by using PyRadiomics and standardized by mean removal and unit variance scaling (Supplementary Table 1 and 2).

### Deep learning modeling

The predictive model was developed using the Python programming language (PyTorch 1.3.1) on a workstation equipped with four NVIDIA RTX3090 data center accelerators (RAM = 24GB). The cascaded multi-modal Transformer architecture consisted of three blocks sequentially integrating features from deep learning features of 3D-MRI, radiological characteristics, and radiomics. This architecture aimed to refine class prediction by integrating and interacting representations of individual modalities^20^. The class prediction process initiated with a learned Query vector Q_**i,**_ progressing through successive blocks while assimilating multi-modal information to generate the final output. Within each block, modalities were transformed into vector embeddings represented as Key vectors K_**e**_ and Value vectors V_**e**_, followed by a cross-attention module to calculate relationships between the modality and previous input sequences. The Dropout module probabilistically determined whether the backbone could receive information in each training iteration, indicating “not available.” Auxiliary output module was used to obtain intermediate output. More details, including input embedding, cascaded cross-attention calculation, dropout module, auxiliary output module, and hyperparameter settings, are provided in Supplementary Note 2.

Dataset A was randomly divided into a training group (*n* = 578) and an internal test group (*n* = 145) at an 8:2 ratio. Dataset B (*n* = 285) was used as the external test group to assess model generalizability. Here we conducted a series of ablation experiments were conducted to determine the best input of each block. Moreover, experiment of setting different combinations of modalities as input was also performed as it could provide valuable insights into which modality was necessary for prediction. Only the model with the best performance was set for subsequent analysis.

Model performance was evaluated using metrics including area under the curve (AUC), accuracy, sensitivity, specificity, F1 score, and Matthews correlation coefficient (MCC). The mean and 95CI (95% confidence interval) of each metrics were calculated based on 50-fold cross-validation. Gradient-weighted class activation mapping (Grad-CAM) was employed to generate heatmaps highlighting important regions based on target class score gradients, along with cross-attention maps to visualized and analyzed with the contributions of each modality to the class prediction.

### Clinical validation

Follow-up asymptomatic small meningioma patients *(Dataset C)* were identified from the Shangjin Hospital between January, 2010 and December, 2017. The inclusion criteria were as follows: (1) radiographic diagnosis of meningioma by brain MRI^5^; (2) asymptomatic lesion; (3) maximal diameter less than 3 cm; (4) without venous sinus involvement; (5) age ≥ 18 years old; (6) with radiological follow-up for at least 5 years. The exclusion criteria were as follows: (1) occurrence of an intervention unrelated to tumor growth (including symptom development, unrelated meningioma-specific mortality, patient request); (2) MR scans with severe motion artifacts; (3) multiple meningiomas; (4) occurrence of irrelevant intracranial diseases. Based on above criteria, a total of 231 cases were accordingly collected.

Survival time was calculated from the date of MR scan to the date of the endpoint, which was defined as fifteen percentage increase in tumor volume in concordance with prior studies^35–37^. Then, tumor growth happened in both 3 years and 5 years were set as ground truth, and predictive metrics of the developed model selected from 2.3.2 were calculated accordingly. There was no other fine tuning or new modeling was performed.

The 231 cases were directly stratified into low-group and high-group based on the prediction of well-trained model. Kaplan–Meier (KM) analysis and log-rank test were used to examine if there was statistical difference between two groups. Moreover, multivariate Cox regression was conducted to investigate if the model prediction was independently associated with patient survival in 5 years. Sub-group analysis was performed to evaluate if the model could achieve similar accuracy in skull-base versus non-skull-base meningiomas.

### Statistical analysis

Categorical variables were described using frequencies and percentages, while continuous variables were summarized using means and standard deviations/95CI. The chi-square test was employed for univariate analysis, and Logistic regression was used for multivariate analysis. *P*-values < 0.05 considered statistically significant.

### Ethics approval and consent to participate

The study was conducted in accordance with the Declaration of Helsinki, and approved by the institutional review board of West China Hospital, Sichuan University (protocol code 2021-S-851). The informed consent was waived by the institutional review board.

## Supplementary information

Supplementary information

## Data Availability

Data is provided within the manuscript or supplementary information files. Due to the privacy of patients, the data related to patients cannot be available for public access but can be obtained from the corresponding author on reasonable request approved by the institutional review board of all enrolled centers. Whereas, we’d like to share the deep learning model publicly once the paper is accepted.
