# Supplementary material for: A multi-modal deep learning model for prediction of Ki-67 for meningiomas using pretreatment MR images
Source: NPJ Precis Oncol. 2025 Jan 21;9:21. doi: 10.1038/s41698-025-00811-1 (PMC11751141; doi:10.1038/s41698-025-00811-1)
Supplement: Supplementary file 1 — Supplementary information [file 41698_2025_811_MOESM1_ESM.pdf]

Supplementary Information

A multi-modal deep learning model for prediction of Ki-67 for meningiomas using pretreatment MR images

Contents

Supplementary Note 1. The scanning protocol of each dataset.

Supplementary Note 2. Implementation details of Transformer-based multimodal representation-learning model.

Supplementary Note 3. The ablation experiment to determine the suitable input of each block.

Supplementary Figure 1. The examples of radiological characteristics evaluation. (A~B) a case with capsular enhancement (red arrow) and cerebral spinal fluid (CSF) cleft surrounding tumor (green arrow). (C~D) a case with intra-tumor necrosis (blue arrow). (E~F) a case with peritumoral edema (pink arrow).

Supplementary Figure 2. Grad-CAM and cross-attention visualization for model interpretation in the following two representative cases. (A~D). A case with a high Ki-67 index. The radiological characteristics of this case include: presence of peritumoral edema (EI > 3), presence of intra-tumor necrosis, absence of cerebral spinal fluid (CSF) cleft surrounding the tumor, and presence of capsular enhancement. The Grad-CAM visualization indicated that the CNN block focused on the tumor areas (B). Additionally, the attention mechanism correctly identified relevant areas (C and D), providing insights into the model's interpretability and decision-making process. (E~H). A case with a low Ki-67 index. The radiological characteristics of this case include: absence of peritumoral edema (EI = 1), absence of intra-tumor necrosis, presence of CSF cleft surrounding the tumor, and presence of capsular enhancement. Despite the CNN block focusing on the tumor areas (F), the cross-attention coefficient of the tumor area was rather low due to the absence of the related radiological characteristics (G and H).

Supplementary Figure 3. ROC curves (A~B) and K-M curves (C~D) that illustrates the results of predicting tumor growth for skull-base versus non-skull-base meningiomas by using the developed model.

Supplementary Table 1. The extracted 1218 radiomics by using Pyradiomics in this research.

Supplementary Table 2. The univariate analysis of the relationship between radiomics features and

Ki-67 index in surgically resected meningioma cases. The t test suggested that 349 of them were statistically different in high Ki-67 group and low Ki-67 group.

Supplementary Table 3. The univariate analysis of the relationship between radiological characteristics and Ki-67 index in surgically resected meningioma cases.

Supplementary Table 4. The multivariate analysis of traditional radiological characteristics by using logistic regression. The tumor volume was stratified based on the optimal cutoff values of 32.9, determined at the point of the maximal Youden index calculated by receiver operating characteristic (ROC) analyses.

Supplementary Table 5. Comparison between the multi-modal learning representing model with existing AI methods for predicting Ki-67 of meningiomas in internal performance.

Supplementary Table 6. Comparison between the multi-modal learning representing model with existing AI methods for predicting Ki-67 of meningiomas in external generalization.

Supplementary Table 7. Univariate and multivariable Cox regression analysis of predictors of tumor volume growth in 5 years.

Supplementary Table 8. Summary of the existing Ki-67 prediction research by using medical image analysis technology.

Supplementary Note 1. The scanning protocol of each dataset.

All the contrast-enhanced MR scans were acquired following the injection of gadopentetate dimeglumine (dose: 0.1 mmol/kg) as the contrast agent. The scanning of dynamic enhanced MRI was conducted within 250 s after injection of the contrast agent.

1.1 The scanning protocol of T1WI

Dataset A: 3.0 T Siemens Trio Scanner system, Slice Thickness=5mm; Repetition Time=1600; Echo Time=9.2s; Echo Number(s)=1; Percent Phase Field of View=71.25; Acquisition Matrix=0\320\148\0; Flip Angle=130 degree.

Dataset B: 3.0T Skyra Scanner system, Slice Thickness=5mm; Repetition Time=1600; Echo Time=8.6s; Echo Number(s)=1; Percent Phase Field of View=84.375; Acquisition Matrix=0\256\175\0; Flip Angle=150 degree.

Dataset C: 3.0 T Siemens TrioTim Scanner system, Slice Thickness=1mm; Repetition Time=1450; Echo Time=1.98s; Echo Number(s)=1; Percent Phase Field of View=90.625; Acquisition Matrix=0\256\232\0; Flip Angle=9 degree.

1.2 The scanning protocol of T2WI

Dataset A: 3.0 T Siemens Trio Scanner system, Slice Thickness=5mm; Repetition Time=4000; Echo Time=93s; Echo Number(s)=1; Percent Phase Field of View=80; Acquisition Matrix=0\320\230\0; Flip Angle=120 degree.

Dataset B: 3.0T Skyra Scanner system, Slice Thickness=5mm; Repetition Time=4000; Echo Time=94s; Echo Number(s)=1; Percent Phase Field of View=100; Acquisition Matrix=0\320\320\0; Flip Angle=150 degree.

Dataset C: 3.0 T Siemens TrioTim Scanner system, Slice Thickness=1mm; Repetition Time=3000; Echo Time=354s; Echo Number(s)=1; Percent Phase Field of View=100; Acquisition Matrix=0\256\258\0; Flip Angle=120 degree.

1.3 The scanning protocol of FLAIR

Dataset A: 3.0 T Siemens Trio Scanner system, Slice Thickness=5mm; Repetition Time=6000; Echo Time=93s; Echo Number(s)=1; Percent Phase Field of View=89.0625; Acquisition Matrix=0\256\198\0; Flip Angle=130 degree.

Dataset B: 3.0T Skyra Scanner system, Slice Thickness=5mm; Repetition Time=5000; Echo Time=85s; Echo Number(s)=1; Percent Phase Field of View=87.5; Acquisition

Matrix=0\256\168\0; Flip Angle=150 degree.

Dataset C: 3.0 T Siemens TrioTim Scanner system, Slice Thickness=1mm; Repetition Time=4000; Echo Time=393s; Echo Number(s)=1; Percent Phase Field of View=100; Acquisition Matrix=0\256\258\0; Flip Angle=120 degree.

1.4 The scanning protocol of T1CE

Dataset A: 3.0 T Siemens Trio Scanner system, Slice Thickness=1mm; Repetition Time=1550; Echo Time=1.98s; Echo Number(s)=1; Percent Phase Field of View=90.625; Acquisition Matrix=0\256\232\0; Flip Angle=9 degree.

Dataset B: 3.0T Skyra Scanner system, Slice Thickness=1 mm; Repetition Time=1550; Echo Time=2.44s; Echo Number(s)=1; Percent Phase Field of View=75; Acquisition Matrix=0\256\154\0; Flip Angle=8 degree.

Dataset C: 3.0 T Siemens TrioTim Scanner system, Slice Thickness=1 mm; Repetition Time=1450; Echo Time=1.98s; Echo Number(s)=1; Percent Phase Field of View=90.625; Acquisition Matrix=0\256\232\0; Flip Angle=9 degree.

Supplementary Note 2. Implementation details of Transformer-based multimodal representation-learning model.

The overview of the multimodal representation-learning network is present as follows:

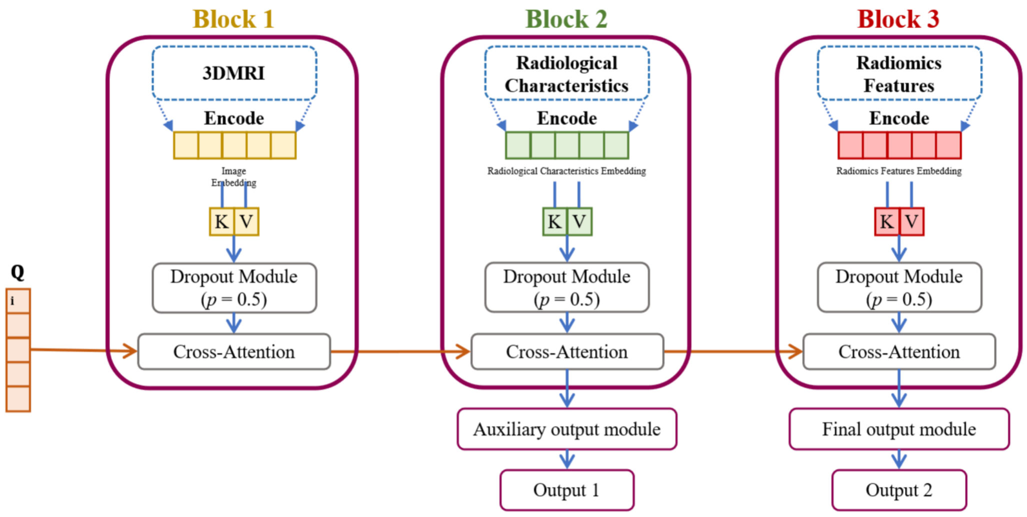

1. Input embedding

1.1 Image embedding

A pretrained CNN-transformer-based feature extractor is employed to capture both local features and global long-range spatial features. The 3D MRI serves as the original input, subsequently undergoing down-sampling via four CNN blocks to generate a high-level feature map, which represents the patch representations of the input containing pertinent information. Position embedding layer is used to preserve the 3D spatial information. Finally, a global average pooling layer and a linear projection layer are utilized to condense the transformer output into a 512-D vector, enabling injection of the imaging feature into the cross-attention module for updating the learned input query.

1.2 Radiological characteristic embedding

The tabular radiological data is transformed into a 512-dimensional one-dimensional vector representation as the input vector by a linear transformation. This linear transformation is performed by a layer of fully connected layers, where the input is a one-dimensional vector and the output is a 512-dimensional embedded vector after the linear transformation.

1.3 Radiomics embedding

The radiomics features is converted to a high-dimensional vector representation through a

linear layer, where the input is also a one-dimensional vector and the output is a mapping from the original one-dimensional vector to the embedding vector learned through the linear layer, just like the radiological characteristic embedding. This embedding is applicable for categorized data.

2. Cascaded cross-attention calculation of the multi-modal data

The blocks comprise a multi-head self-attention layer and a multi-head cross-attention layer, as depicted as following.

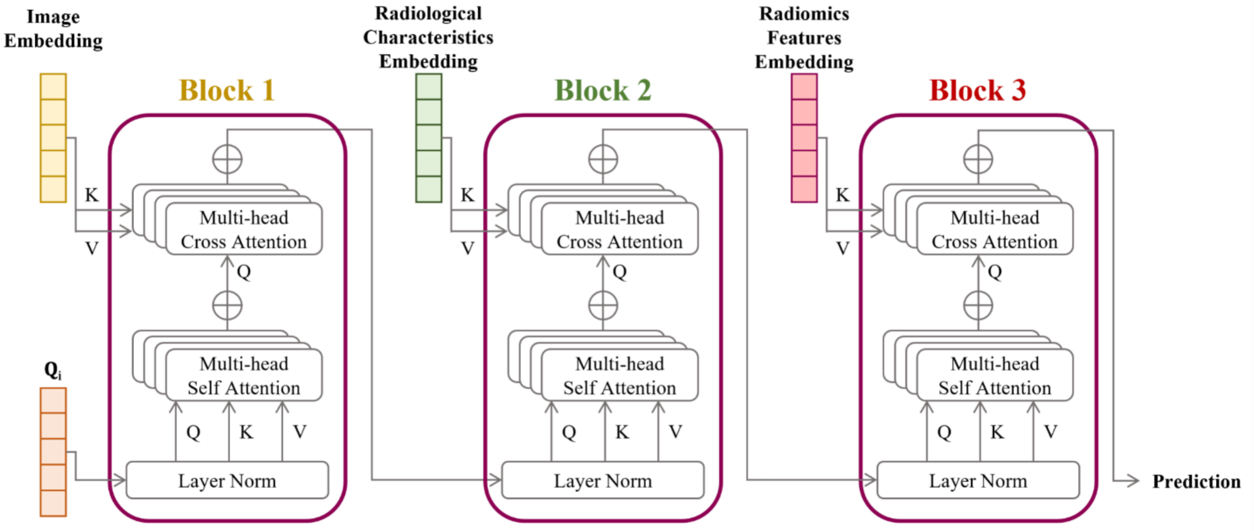

Within the multi-head self-attention layer, queries, keys, and values are denoted as **Q**, **K**, and **V**, respectively. The self-attention dot product matrix can be defined as follows:

$$\boldsymbol{Attention}\,(\boldsymbol{Q},\boldsymbol{K},\boldsymbol{V}) = Softmax\left(\frac{\boldsymbol{QK}^T}{\sqrt{d_k}}\right)\boldsymbol{V} \tag{1}$$

And the multi-head self-attention can be presented as follows:

$$\boldsymbol{head_i} = Attention\left(\boldsymbol{QW}_i^Q, \boldsymbol{KW}_i^K, \boldsymbol{V}_i^V\right) \tag{2}$$

$$\boldsymbol{Multihead}\,(\boldsymbol{Q},\boldsymbol{K},\boldsymbol{V}) = concat(head_1, \dots \dots, head_8)W \tag{3}$$

where $d_k = 512$ in our research.

The cross-attention layer operates similarly to the self-attention layer, with the distinction that instead of computing attention within itself, cross-attention calculates attention between inputs. Define as follows:

$$\boldsymbol{Attention}\ (\boldsymbol{Q}, \boldsymbol{K}, \boldsymbol{V}) = Softmax\left(\frac{\boldsymbol{Q}\boldsymbol{K}_{\boldsymbol{e}}^{\boldsymbol{T}}}{\sqrt{d_k}}\right)\boldsymbol{V}_{\boldsymbol{e}} \quad (4)$$

The modality (MRI, radiological characteristics, or radiomics) embeddings **e** was projected as $\mathbf{K_e}$ and $\mathbf{V_e}$ in each block, respectively. As transformers maintained the shape of the **Q** throughout, multiple blocks can be cascaded to inject mixed multi-modal information into the initial query, followed by cross-attention calculation of **Q** and $\mathbf{K_e}$ and $\mathbf{V_e}$. So, the final output can represent as the refined result after processing all inputs.

This design facilitated the independent integration of each input modality into the network architecture, ensuring the model's functionality remains robust even in instances where specific modalities are absent.

3. Dropout module

The term "dropout" refers to dropping out modality in this epoch of training to simulate missing data scenarios. The parameter p, denoting the dropout rate, can be adjusted for each modality's auxiliary outputs, with a default value typically set at 0.5. It should be noted that selection of p is contingent upon the severity of missing data instances, necessitating careful consideration to optimize model performance under varying conditions of data incompleteness.

4. Auxiliary output module

The auxiliary output module consists of two densely connected layers. The first fully connected layer inputs and outputs are both 512-dimensional vectors, and the second fully connected layer accepts the 512-dimensional vectors from the output of the first fully connected layer, and uses 2 output neurons to adjust the output dimensions to the number of classifications. A LeakyReLU activation function is applied after the first densely connected layer. It's noteworthy that auxiliary outputs are disregarded during inference, and there is no weight sharing between the auxiliary classifier and the final classifier.

5. Training hyperparameter settings

After balancing the model performance with compute capability, hyperparameters of the network are set as follows: the input image size is set to 128 × 128 × 128 with spatial normalization. An SGD optimizer is used with the initial learning rate set to 3×10^{-5}, and the weights decay for L2 normalization is 1×10^{-3} to prevent network overfitting. Size of min-batch is 16, containing 8 samples from source and 8 from target. The final process matches source and target data loaders to eliminate the binding of samples from source and target in one min-batch. The blocks for processing the image modality are set up as 4 self-attention modules as well as 4 cross-attention modules. For Radiological characteristic, 2 self-attention modules as well as 3 cross-attention modules are used. Finally, we used 3 self-attention modules as well as 4 cross-attention modules for Radiomics. At the same time, we initialized the potential query vector as a one-dimensional vector with a sequence length of 128 dimensions obeying a Gaussian distribution.

Supplementary Note 3. The ablation experiment to determine the suitable input of each block.

1. 3D-MRIs block

In most previous studies, the segmentation-classification pipeline is the most popular method, as it simultaneously enhances accuracy and leads to faster model convergence during training by eliminating irrelevant information, simplifies input data, and reduces computational demands. However, as suggested in ***Supplemental Table 3***, the Edema Index (EI) is statistically related to Ki-67, indicating that the peritumor area may also contain critical information important for accurate classification. Additionally, given that multimodal learning requires interaction between 3D-MRIs and radiological characteristics, determining the best input for this block is crucial for optimizing performance.

Generally, there are three alternatives for model input, including whole MRI series, segmented tumor, and central-cropped MRI. For the centrally-cropped MRI images, the tumor masks were defined as the central region, extending outward by 10 voxels. All images were resampled to a resolution of 128x128x128 for model input. The results are presented as follows:

**Input**	**AUC**	**Sensitivity**	**Specificity**
**Segmented tumor**	0.594	0.469	0.625
**whole MRI series**	0.528	0.122	0.750
**Central-cropped MRI**	0.765	0.224	0.875

2. Radiomics block

Although radiomic features serve as the quantitative analysis of MRI, their high-dimensional and redundant nature poses challenges for deep learning. As indicated in ***Supplementary Material 3***, only 349 out of the 1218 extracted features exhibited statistical significance. Consequently, we used both the complete set of extracted features and the subset of selected features as input for ablation experiments. The results are presented as follows:

**Input**	**AUC**	**Sensitivity**	**Specificity**
**All extracted features**	0.765	0.224	0.875
**Selected features**	0.785	0.736	0.764

Above all, based on the results of experiment, the best input for 3D-MRIs block is the Central-cropped MRI, and for radiomics block is the statistically related feature. Please note that cross validation was not performed in the ablation experiment.

Supplementary Figure 1.

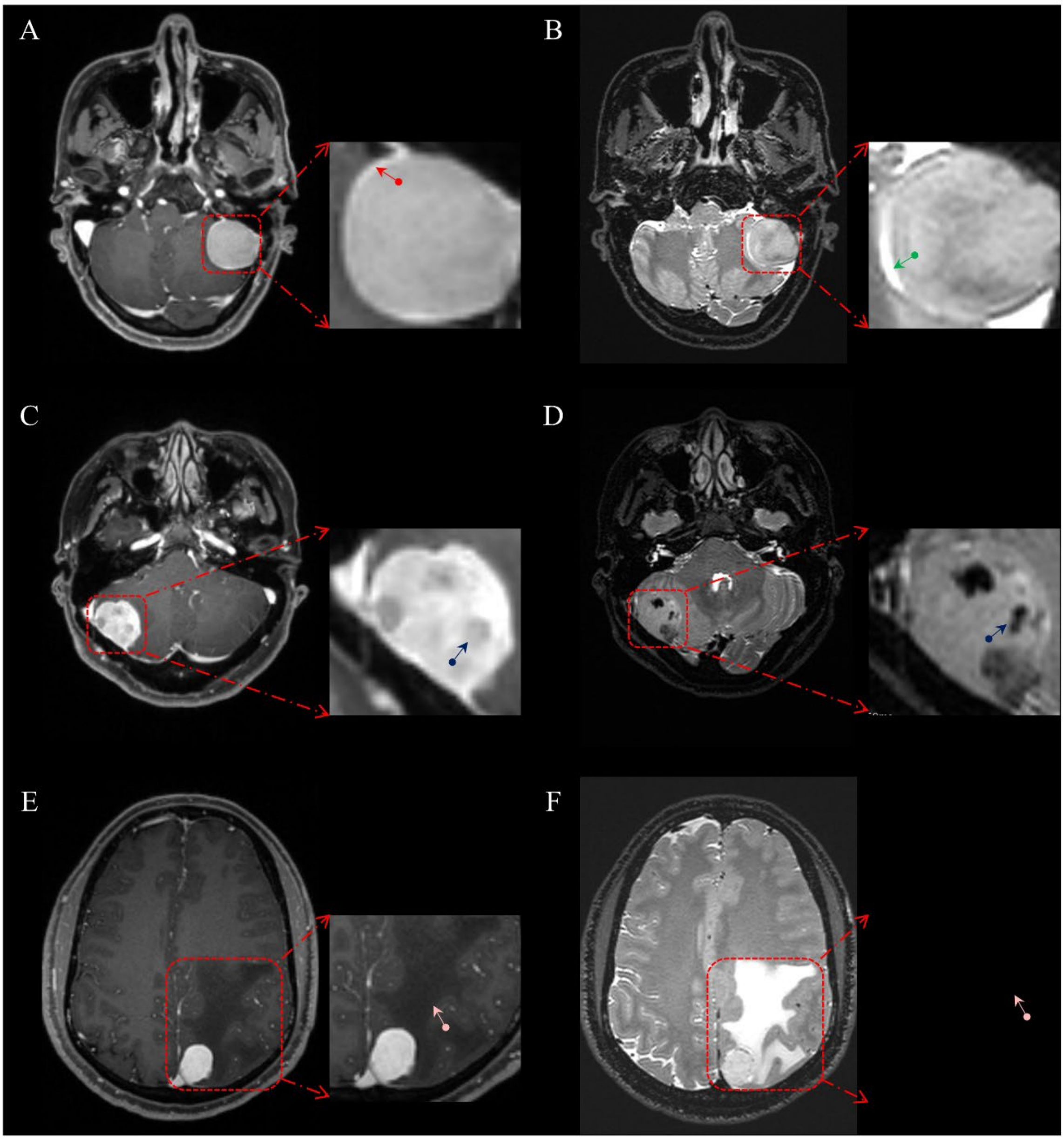

The examples of radiological characteristics evaluation. (A~B) a case with capsular enhancement (red arrow) and cerebral spinal fluid (CSF) cleft surrounding tumor (green arrow). (C~D) a case with intra-tumor necrosis (blue arrow). (E~F) a case with peritumoral edema (pink arrow).

Supplementary Figure 2. Grad-CAM and cross-attention visualization for model interpretation in the following two representative cases.

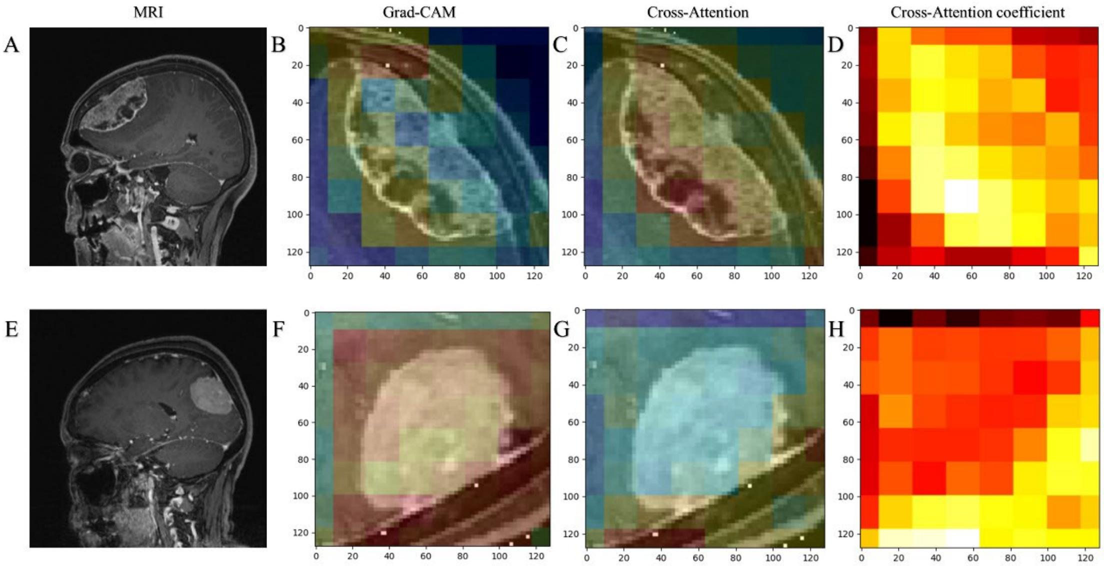

(A~D). A case with a high Ki-67 index. The radiological characteristics of this case include: presence of peritumoral edema (EI > 3), presence of intra-tumor necrosis, absence of cerebral spinal fluid (CSF) cleft surrounding the tumor, and presence of capsular enhancement. The Grad-CAM visualization indicated that the CNN block focused on the tumor areas (B). Additionally, the attention mechanism correctly identified relevant areas (C and D), providing insights into the model's interpretability and decision-making process. (E~H). A case with a low Ki-67 index. The radiological characteristics of this case include: absence of peritumoral edema (EI = 1), absence of intra-tumor necrosis, presence of CSF cleft surrounding the tumor, and presence of capsular enhancement. Despite the CNN block focusing on the tumor areas (F), the cross-attention coefficient of the tumor area was rather low due to the absence of the related radiological characteristics (G and H).

Supplementary Figure 3.

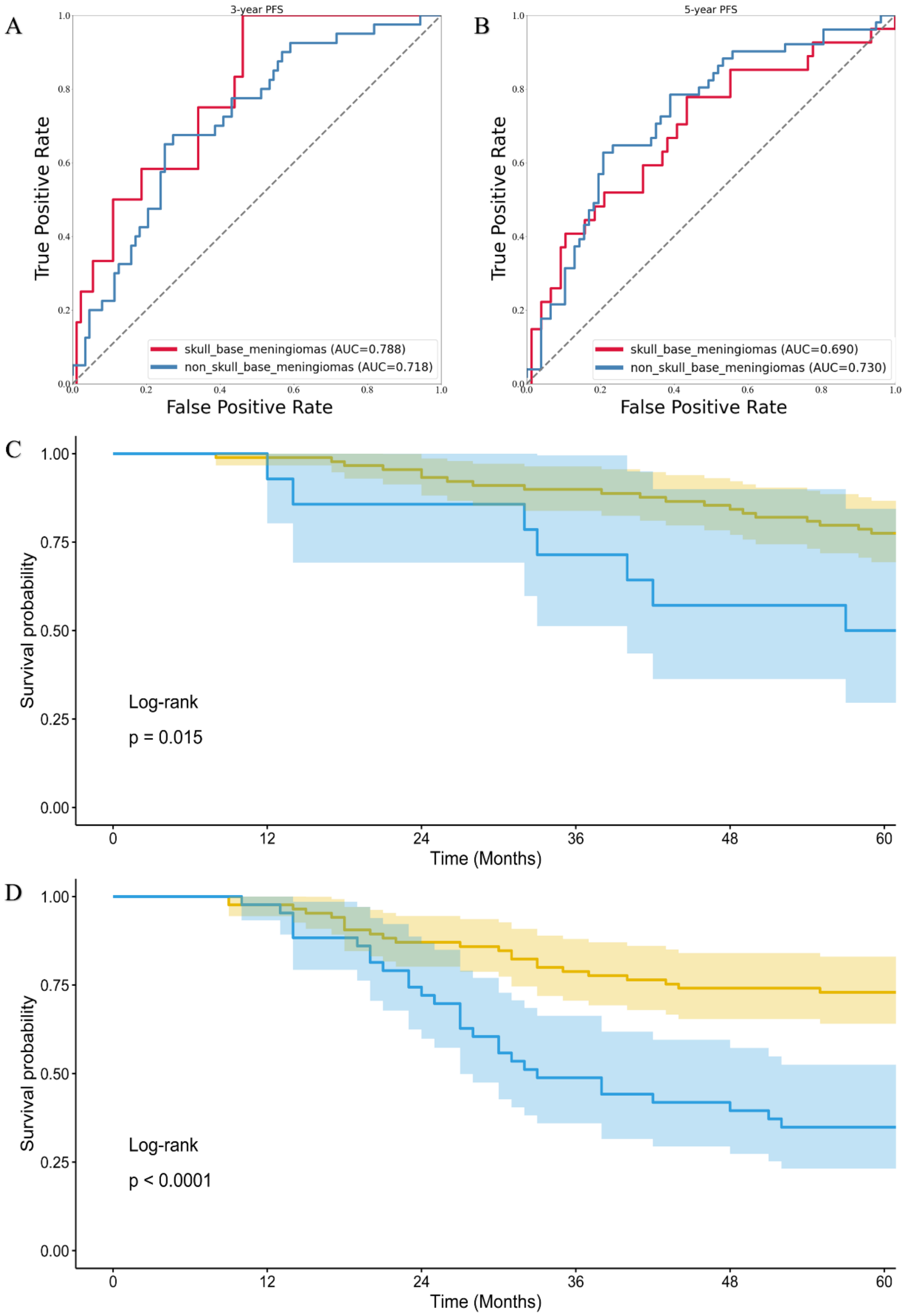

ROC curves (A~B) and K-M curves (C~D) that illustrates the results of predicting tumor growth for skull-base versus non-skull-base meningiomas by using the developed model.

Supplementary Table 1. The extracted 1218 radiomics by using Pyradiomics in this research.

Matrix	Filter	Features
Shape	Original	Elongation
		Flatness
		LeastAxisLength
		MajorAxisLength
		Maximum2DDiameterColumn
		Maximum2DDiameterRow
		Maximum2DDiameterSlice
		Maximum3DDiameter
		MeshVolume
		MinorAxisLength
		Sphericity
		SurfaceArea
		SurfaceVolumeRatio
		VoxelVolume
First Order	original	10Percentile
	log-sigma-1-0-mm-3D	90Percentile
	log-sigma-2-0-mm-3D	Energy
	log-sigma-3-0-mm-3D	Entropy
	log-sigma-4-0-mm-3D	InterquartileRange
	log-sigma-5-0-mm-3D	Kurtosis
	wavelet-LLH	Maximum
	wavelet-LHL	MeanAbsoluteDeviation
	wavelet-LHH	Mean
	wavelet-HLL	Median
	wavelet-HLH	Minimum
	wavelet-HHL	Range
	wavelet-HHH	RobustMeanAbsoluteDeviation
	wavelet-LLL	RootMeanSquared
		Skewness
		TotalEnergy
		Uniformity
		Variance
GLCM	original	Autocorrelation
	log-sigma-1-0-mm-3D	JointAverage
	log-sigma-2-0-mm-3D	ClusterProminence
	log-sigma-3-0-mm-3D	ClusterShade
	log-sigma-4-0-mm-3D	ClusterTendency
	log-sigma-5-0-mm-3D	Contrast
	wavelet-LLH	Correlation
	wavelet-LHL	DifferenceAverage

	wavelet-LHH	DifferenceEntropy
	wavelet-HLL	DifferenceVariance
	wavelet-HLH	JointEnergy
	wavelet-HHL	JointEntropy
	wavelet-HHH	Imc1
	wavelet-LLL	Imc2
		Idm
		Idmn
		Id
		Idn
		InverseVariance
		MaximumProbability
		SumEntropy
		SumSquares
GLRLM	original	GrayLevelNonUniformity
	log-sigma-1-0-mm-3D	GrayLevelNonUniformityNormalized
	log-sigma-2-0-mm-3D	GrayLevelVariance
	log-sigma-3-0-mm-3D	HighGrayLevelRunEmphasis
	log-sigma-4-0-mm-3D	LongRunEmphasis
	log-sigma-5-0-mm-3D	LongRunHighGrayLevelEmphasis
	wavelet-LLH	LongRunLowGrayLevelEmphasis
	wavelet-LHL	LowGrayLevelRunEmphasis
	wavelet-LHH	RunEntropy
	wavelet-HLL	RunLengthNonUniformity
	wavelet-HLH	RunLengthNonUniformityNormalized
	wavelet-HHL	RunPercentage
	wavelet-HHH	RunVariance
	wavelet-LLL	ShortRunEmphasis
		ShortRunHighGrayLevelEmphasis
		ShortRunLowGrayLevelEmphasis
GLSZM	original	GrayLevelNonUniformity
	log-sigma-1-0-mm-3D	GrayLevelNonUniformityNormalized
	log-sigma-2-0-mm-3D	GrayLevelVariance
	log-sigma-3-0-mm-3D	HighGrayLevelZoneEmphasis
	log-sigma-4-0-mm-3D	LargeAreaEmphasis
	log-sigma-5-0-mm-3D	LargeAreaHighGrayLevelEmphasis
	wavelet-LLH	LargeAreaLowGrayLevelEmphasis
	wavelet-LHL	LowGrayLevelZoneEmphasis
	wavelet-LHH	SizeZoneNonUniformity
	wavelet-HLL	SizeZoneNonUniformityNormalized
	wavelet-HLH	SmallAreaEmphasis
	wavelet-HHL	SmallAreaHighGrayLevelEmphasis
	wavelet-HHH	SmallAreaLowGrayLevelEmphasis
	wavelet-LLL	ZoneEntropy

		ZonePercentage<br>ZoneVariance
GLDM	original	DependenceEntropy
	log-sigma-1-0-mm-3D	DependenceNonUniformity
	log-sigma-2-0-mm-3D	DependenceNonUniformityNormalized
	log-sigma-3-0-mm-3D	DependenceVariance
	log-sigma-4-0-mm-3D	GrayLevelNonUniformity
	log-sigma-5-0-mm-3D	GrayLevelVariance
	wavelet-LLH	HighGrayLevelEmphasis
	wavelet-LHL	LargeDependenceEmphasis
	wavelet-LHH	LargeDependenceHighGrayLevelEmphasis
	wavelet-HLL	LargeDependenceLowGrayLevelEmphasis
	wavelet-HLH	LowGrayLevelEmphasis
	wavelet-HHL	SmallDependenceEmphasis
	wavelet-HHH	SmallDependenceHighGrayLevelEmphasis
	wavelet-LLL	SmallDependenceLowGrayLevelEmphasis

GLCM: Gray-level co-occurrence matrix; GLRLM: Gray-level run length matrix; GLSZM: Gray-level size zone matrix; GLDM: Gray-level dependence matrix.

The t test suggested that 349 of them were statistically different in high Ki-67 group and low Ki-67 group, shown as follows:

**Radiomics Feature**	**p**
original_shape_SurfaceVolumeRatio	0.024
original_firstorder_Median	0.038
original_firstorder_Skewness	0.000
original_glcm_Imc1	0.037
original_glcm_Idmn	0.024
original_glrlm_GrayLevelNonUniformity	0.002
original_glszm_SizeZoneNonUniformityNormalized	0.003
original_glszm_SmallAreaEmphasis	0.003
original_gldm_GrayLevelNonUniformity	0.015
original_gldm_LargeDependenceHighGrayLevelEmphasis	0.006
log_sigma_1_0_mm_3D_firstorder_Kurtosis	0.033
log_sigma_1_0_mm_3D_firstorder_Mean	0.034
log_sigma_1_0_mm_3D_firstorder_Median	0.041
log_sigma_1_0_mm_3D_firstorder_RootMeanSquared	0.027
log_sigma_1_0_mm_3D_glcm_Autocorrelation	0.042
log_sigma_1_0_mm_3D_glcm_JointAverage	0.030
log_sigma_1_0_mm_3D_glcm_Imc1	0.019
log_sigma_1_0_mm_3D_glcm_Imc2	0.009
log_sigma_1_0_mm_3D_glcm_Idmn	0.025
log_sigma_1_0_mm_3D_glcm_Idn	0.030
log_sigma_1_0_mm_3D_glrlm_GrayLevelNonUniformity	0.002

log_sigma_1_0_mm_3D_glrlm_LongRunHighGrayLevelEmphasis	0.044
log_sigma_1_0_mm_3D_glszm_SizeZoneNonUniformityNormalized	0.016
log_sigma_1_0_mm_3D_glszm_SmallAreaEmphasis	0.013
log_sigma_1_0_mm_3D_glszm_ZoneEntropy	0.030
log_sigma_1_0_mm_3D_gldm_GrayLevelNonUniformity	0.017
log_sigma_2_0_mm_3D_firstorder_10Percentile	0.017
log_sigma_2_0_mm_3D_firstorder_InterquartileRange	0.040
log_sigma_2_0_mm_3D_firstorder_Kurtosis	0.030
log_sigma_2_0_mm_3D_firstorder_Mean	0.002
log_sigma_2_0_mm_3D_firstorder_Median	0.001
log_sigma_2_0_mm_3D_firstorder_RootMeanSquared	0.002
log_sigma_2_0_mm_3D_firstorder_Skewness	0.010
log_sigma_2_0_mm_3D_glcm_Autocorrelation	0.003
log_sigma_2_0_mm_3D_glcm_JointAverage	0.003
log_sigma_2_0_mm_3D_glcm_Correlation	0.005
log_sigma_2_0_mm_3D_glcm_DifferenceAverage	0.018
log_sigma_2_0_mm_3D_glcm_DifferenceEntropy	0.026
log_sigma_2_0_mm_3D_glcm_Imc1	0.004
log_sigma_2_0_mm_3D_glcm_Imc2	0.021
log_sigma_2_0_mm_3D_glcm_Idm	0.015
log_sigma_2_0_mm_3D_glcm_Idmn	0.005
log_sigma_2_0_mm_3D_glcm_Id	0.015
log_sigma_2_0_mm_3D_glcm_Idn	0.001
log_sigma_2_0_mm_3D_glcm_InverseVariance	0.004
log_sigma_2_0_mm_3D_glrlm_GrayLevelNonUniformity	0.001
log_sigma_2_0_mm_3D_glrlm_HighGrayLevelRunEmphasis	0.005
log_sigma_2_0_mm_3D_glrlm_LongRunHighGrayLevelEmphasis	0.005
log_sigma_2_0_mm_3D_glrlm_RunLengthNonUniformityNormalized	0.012
log_sigma_2_0_mm_3D_glrlm_RunPercentage	0.035
log_sigma_2_0_mm_3D_glrlm_ShortRunEmphasis	0.019
log_sigma_2_0_mm_3D_glrlm_ShortRunHighGrayLevelEmphasis	0.009
log_sigma_2_0_mm_3D_glszm_HighGrayLevelZoneEmphasis	0.018
log_sigma_2_0_mm_3D_glszm_SizeZoneNonUniformity	0.001
log_sigma_2_0_mm_3D_glszm_SizeZoneNonUniformityNormalized	0.024
log_sigma_2_0_mm_3D_glszm_SmallAreaEmphasis	0.021
log_sigma_2_0_mm_3D_glszm_ZoneEntropy	0.033
log_sigma_2_0_mm_3D_glszm_ZonePercentage	0.006
log_sigma_2_0_mm_3D_gldm_DependenceEntropy	0.024
log_sigma_2_0_mm_3D_gldm_DependenceNonUniformityNormalized	0.010
log_sigma_2_0_mm_3D_gldm_GrayLevelNonUniformity	0.008
log_sigma_2_0_mm_3D_gldm_HighGrayLevelEmphasis	0.004
log_sigma_2_0_mm_3D_gldm_LargeDependenceHighGrayLevelEmphasis	0.005
log_sigma_2_0_mm_3D_gldm_SmallDependenceEmphasis	0.006
log_sigma_3_0_mm_3D_firstorder_10Percentile	0.004

log_sigma_3_0_mm_3D_firstorder_90Percentile	0.003
log_sigma_3_0_mm_3D_firstorder_Mean	0.000
log_sigma_3_0_mm_3D_firstorder_Median	0.000
log_sigma_3_0_mm_3D_firstorder_RootMeanSquared	0.000
log_sigma_3_0_mm_3D_firstorder_Skewness	0.019
log_sigma_3_0_mm_3D_glcm_Contrast	0.015
log_sigma_3_0_mm_3D_glcm_Correlation	0.000
log_sigma_3_0_mm_3D_glcm_DifferenceAverage	0.005
log_sigma_3_0_mm_3D_glcm_DifferenceEntropy	0.005
log_sigma_3_0_mm_3D_glcm_DifferenceVariance	0.018
log_sigma_3_0_mm_3D_glcm_Imc2	0.000
log_sigma_3_0_mm_3D_glcm_Idm	0.004
log_sigma_3_0_mm_3D_glcm_Idmn	0.018
log_sigma_3_0_mm_3D_glcm_Id	0.004
log_sigma_3_0_mm_3D_glcm_Idn	0.004
log_sigma_3_0_mm_3D_glcm_InverseVariance	0.002
log_sigma_3_0_mm_3D_glrlm_GrayLevelNonUniformity	0.000
log_sigma_3_0_mm_3D_glrlm_RunLengthNonUniformityNormalized	0.003
log_sigma_3_0_mm_3D_glrlm_RunPercentage	0.007
log_sigma_3_0_mm_3D_glrlm_ShortRunEmphasis	0.005
log_sigma_3_0_mm_3D_glszm_GrayLevelNonUniformity	0.000
log_sigma_3_0_mm_3D_glszm_SizeZoneNonUniformity	0.012
log_sigma_3_0_mm_3D_glszm_SizeZoneNonUniformityNormalized	0.028
log_sigma_3_0_mm_3D_glszm_SmallAreaEmphasis	0.031
log_sigma_3_0_mm_3D_glszm_ZonePercentage	0.003
log_sigma_3_0_mm_3D_gldm_DependenceEntropy	0.006
log_sigma_3_0_mm_3D_gldm_DependenceNonUniformityNormalized	0.007
log_sigma_3_0_mm_3D_gldm_DependenceVariance	0.037
log_sigma_3_0_mm_3D_gldm_GrayLevelNonUniformity	0.005
log_sigma_3_0_mm_3D_gldm_LargeDependenceEmphasis	0.026
log_sigma_3_0_mm_3D_gldm_LargeDependenceHighGrayLevelEmphasis	0.027
log_sigma_3_0_mm_3D_gldm_SmallDependenceEmphasis	0.003
log_sigma_4_0_mm_3D_firstorder_10Percentile	0.001
log_sigma_4_0_mm_3D_firstorder_90Percentile	0.001
log_sigma_4_0_mm_3D_firstorder_InterquartileRange	0.020
log_sigma_4_0_mm_3D_firstorder_MeanAbsoluteDeviation	0.046
log_sigma_4_0_mm_3D_firstorder_Mean	0.000
log_sigma_4_0_mm_3D_firstorder_RobustMeanAbsoluteDeviation	0.026
log_sigma_4_0_mm_3D_glcm_Contrast	0.004
log_sigma_4_0_mm_3D_glcm_Correlation	0.001
log_sigma_4_0_mm_3D_glcm_DifferenceAverage	0.002
log_sigma_4_0_mm_3D_glcm_DifferenceEntropy	0.001
log_sigma_4_0_mm_3D_glcm_DifferenceVariance	0.002
log_sigma_4_0_mm_3D_glcm_JointEntropy	0.034

log_sigma_4_0_mm_3D_glcm_Imc2	0.001
log_sigma_4_0_mm_3D_glcm_Idm	0.002
log_sigma_4_0_mm_3D_glcm_Idmn	0.010
log_sigma_4_0_mm_3D_glcm_Id	0.002
log_sigma_4_0_mm_3D_glcm_Idn	0.002
log_sigma_4_0_mm_3D_glcm_InverseVariance	0.001
log_sigma_4_0_mm_3D_glrlm_GrayLevelNonUniformity	0.000
log_sigma_4_0_mm_3D_glrlm_LongRunEmphasis	0.035
log_sigma_4_0_mm_3D_glrlm_RunLengthNonUniformityNormalized	0.002
log_sigma_4_0_mm_3D_glrlm_RunPercentage	0.003
log_sigma_4_0_mm_3D_glrlm_ShortRunEmphasis	0.003
log_sigma_4_0_mm_3D_glszm_GrayLevelNonUniformity	0.003
log_sigma_4_0_mm_3D_glszm_SizeZoneNonUniformityNormalized	0.018
log_sigma_4_0_mm_3D_glszm_SmallAreaEmphasis	0.027
log_sigma_4_0_mm_3D_glszm_ZonePercentage	0.001
log_sigma_4_0_mm_3D_gldm_DependenceEntropy	0.017
log_sigma_4_0_mm_3D_gldm_DependenceNonUniformityNormalized	0.008
log_sigma_4_0_mm_3D_gldm_DependenceVariance	0.023
log_sigma_4_0_mm_3D_gldm_GrayLevelNonUniformity	0.003
log_sigma_4_0_mm_3D_gldm_LargeDependenceEmphasis	0.013
log_sigma_4_0_mm_3D_gldm_LargeDependenceHighGrayLevelEmphasis	0.040
log_sigma_4_0_mm_3D_gldm_SmallDependenceEmphasis	0.001
log_sigma_5_0_mm_3D_firstorder_10Percentile	0.000
log_sigma_5_0_mm_3D_firstorder_90Percentile	0.002
log_sigma_5_0_mm_3D_firstorder_Entropy	0.014
log_sigma_5_0_mm_3D_firstorder_InterquartileRange	0.001
log_sigma_5_0_mm_3D_firstorder_Kurtosis	0.011
log_sigma_5_0_mm_3D_firstorder_MeanAbsoluteDeviation	0.004
log_sigma_5_0_mm_3D_firstorder_Minimum	0.044
log_sigma_5_0_mm_3D_firstorder_RobustMeanAbsoluteDeviation	0.001
log_sigma_5_0_mm_3D_firstorder_Uniformity	0.013
log_sigma_5_0_mm_3D_firstorder_Variance	0.026
log_sigma_5_0_mm_3D_glcm_Contrast	0.001
log_sigma_5_0_mm_3D_glcm_Correlation	0.026
log_sigma_5_0_mm_3D_glcm_DifferenceAverage	0.000
log_sigma_5_0_mm_3D_glcm_DifferenceEntropy	0.000
log_sigma_5_0_mm_3D_glcm_DifferenceVariance	0.001
log_sigma_5_0_mm_3D_glcm_JointEnergy	0.029
log_sigma_5_0_mm_3D_glcm_JointEntropy	0.006
log_sigma_5_0_mm_3D_glcm_Imc1	0.000
log_sigma_5_0_mm_3D_glcm_Idm	0.000
log_sigma_5_0_mm_3D_glcm_Idmn	0.005
log_sigma_5_0_mm_3D_glcm_Id	0.000
log_sigma_5_0_mm_3D_glcm_Idn	0.001

log_sigma_5_0_mm_3D_glcm_InverseVariance	0.000
log_sigma_5_0_mm_3D_glcm_SumEntropy	0.033
log_sigma_5_0_mm_3D_glrlm_GrayLevelNonUniformity	0.000
log_sigma_5_0_mm_3D_glrlm_GrayLevelNonUniformityNormalized	0.016
log_sigma_5_0_mm_3D_glrlm_GrayLevelVariance	0.028
log_sigma_5_0_mm_3D_glrlm_LongRunEmphasis	0.013
log_sigma_5_0_mm_3D_glrlm_RunLengthNonUniformityNormalized	0.000
log_sigma_5_0_mm_3D_glrlm_RunPercentage	0.001
log_sigma_5_0_mm_3D_glrlm_RunVariance	0.019
log_sigma_5_0_mm_3D_glrlm_ShortRunEmphasis	0.001
log_sigma_5_0_mm_3D_glszm_GrayLevelNonUniformity	0.023
log_sigma_5_0_mm_3D_glszm_ZonePercentage	0.000
log_sigma_5_0_mm_3D_gldm_DependenceEntropy	0.018
log_sigma_5_0_mm_3D_gldm_DependenceNonUniformityNormalized	0.000
log_sigma_5_0_mm_3D_gldm_DependenceVariance	0.006
log_sigma_5_0_mm_3D_gldm_GrayLevelNonUniformity	0.002
log_sigma_5_0_mm_3D_gldm_GrayLevelVariance	0.026
log_sigma_5_0_mm_3D_gldm_LargeDependenceEmphasis	0.004
log_sigma_5_0_mm_3D_gldm_LargeDependenceHighGrayLevelEmphasis	0.011
log_sigma_5_0_mm_3D_gldm_SmallDependenceEmphasis	0.000
log_sigma_5_0_mm_3D_gldm_SmallDependenceHighGrayLevelEmphasis	0.024
log_sigma_5_0_mm_3D_gldm_SmallDependenceLowGrayLevelEmphasis	0.049
wavelet_LLH_firstorder_90Percentile	0.047
wavelet_LLH_firstorder_Maximum	0.000
wavelet_LLH_firstorder_Mean	0.007
wavelet_LLH_firstorder_Minimum	0.029
wavelet_LLH_firstorder_Range	0.003
wavelet_LLH_firstorder_RootMeanSquared	0.000
wavelet_LLH_glcm_Autocorrelation	0.029
wavelet_LLH_glcm_JointAverage	0.017
wavelet_LLH_glcm_Correlation	0.001
wavelet_LLH_glcm_Imc1	0.040
wavelet_LLH_glcm_Imc2	0.004
wavelet_LLH_glrlm_GrayLevelNonUniformity	0.006
wavelet_LLH_glrlm_HighGrayLevelRunEmphasis	0.029
wavelet_LLH_glrlm_LongRunHighGrayLevelEmphasis	0.035
wavelet_LLH_glrlm_ShortRunHighGrayLevelEmphasis	0.029
wavelet_LLH_glszm_HighGrayLevelZoneEmphasis	0.024
wavelet_LLH_glszm_SmallAreaHighGrayLevelEmphasis	0.038
wavelet_LLH_gldm_GrayLevelNonUniformity	0.025
wavelet_LLH_gldm_HighGrayLevelEmphasis	0.029
wavelet_LHL_firstorder_Maximum	0.034
wavelet_LHL_firstorder_Minimum	0.043
wavelet_LHL_firstorder_Range	0.032

wavelet_LHL_firstorder_RootMeanSquared	0.014
wavelet_LHL_glcm_Autocorrelation	0.029
wavelet_LHL_glcm_JointAverage	0.033
wavelet_LHL_glcm_Correlation	0.001
wavelet_LHL_glcm_Imc1	0.006
wavelet_LHL_glcm_Imc2	0.000
wavelet_LHL_glrlm_GrayLevelNonUniformity	0.005
wavelet_LHL_glrlm_HighGrayLevelRunEmphasis	0.030
wavelet_LHL_glrlm_LongRunHighGrayLevelEmphasis	0.044
wavelet_LHL_glrlm_ShortRunHighGrayLevelEmphasis	0.029
wavelet_LHL_glszm_HighGrayLevelZoneEmphasis	0.028
wavelet_LHL_glszm_SmallAreaHighGrayLevelEmphasis	0.038
wavelet_LHL_gldm_GrayLevelNonUniformity	0.015
wavelet_LHL_gldm_HighGrayLevelEmphasis	0.030
wavelet_LHL_gldm_SmallDependenceHighGrayLevelEmphasis	0.042
wavelet_LHH_firstorder_10Percentile	0.039
wavelet_LHH_firstorder_InterquartileRange	0.025
wavelet_LHH_firstorder_Maximum	0.016
wavelet_LHH_firstorder_Minimum	0.012
wavelet_LHH_firstorder_Range	0.012
wavelet_LHH_firstorder_RobustMeanAbsoluteDeviation	0.031
wavelet_LHH_glcm_Autocorrelation	0.013
wavelet_LHH_glcm_JointAverage	0.010
wavelet_LHH_glcm_Contrast	0.042
wavelet_LHH_glcm_Correlation	0.000
wavelet_LHH_glcm_DifferenceAverage	0.030
wavelet_LHH_glcm_JointEntropy	0.047
wavelet_LHH_glcm_Imc1	0.002
wavelet_LHH_glcm_Imc2	0.001
wavelet_LHH_glcm_Idm	0.021
wavelet_LHH_glcm_Id	0.022
wavelet_LHH_glcm_InverseVariance	0.021
wavelet_LHH_glcm_MaximumProbability	0.030
wavelet_LHH_glrlm_GrayLevelNonUniformity	0.002
wavelet_LHH_glrlm_HighGrayLevelRunEmphasis	0.013
wavelet_LHH_glrlm_LongRunHighGrayLevelEmphasis	0.031
wavelet_LHH_glrlm_ShortRunHighGrayLevelEmphasis	0.012
wavelet_LHH_glszm_HighGrayLevelZoneEmphasis	0.014
wavelet_LHH_glszm_SmallAreaHighGrayLevelEmphasis	0.017
wavelet_LHH_gldm_DependenceNonUniformityNormalized	0.027
wavelet_LHH_gldm_DependenceVariance	0.009
wavelet_LHH_gldm_GrayLevelNonUniformity	0.010
wavelet_LHH_gldm_HighGrayLevelEmphasis	0.013
wavelet_LHH_gldm_LargeDependenceEmphasis	0.047

wavelet_LHH_gldm_SmallDependenceHighGrayLevelEmphasis	0.026
wavelet_HLL_firstorder_Maximum	0.033
wavelet_HLL_firstorder_Mean	0.002
wavelet_HLL_firstorder_Median	0.022
wavelet_HLL_firstorder_Minimum	0.047
wavelet_HLL_firstorder_Range	0.031
wavelet_HLL_firstorder_RootMeanSquared	0.000
wavelet_HLL_glcm_Autocorrelation	0.023
wavelet_HLL_glcm_JointAverage	0.034
wavelet_HLL_glcm_Correlation	0.016
wavelet_HLL_glcm_Imc1	0.008
wavelet_HLL_glcm_Imc2	0.001
wavelet_HLL_glrlm_GrayLevelNonUniformity	0.009
wavelet_HLL_glrlm_HighGrayLevelRunEmphasis	0.024
wavelet_HLL_glrlm_LongRunHighGrayLevelEmphasis	0.038
wavelet_HLL_glrlm_ShortRunHighGrayLevelEmphasis	0.023
wavelet_HLL_glszm_HighGrayLevelZoneEmphasis	0.023
wavelet_HLL_glszm_SmallAreaHighGrayLevelEmphasis	0.028
wavelet_HLL_gldm_GrayLevelNonUniformity	0.029
wavelet_HLL_gldm_HighGrayLevelEmphasis	0.024
wavelet_HLL_gldm_SmallDependenceHighGrayLevelEmphasis	0.032
wavelet_HLH_firstorder_Maximum	0.045
wavelet_HLH_firstorder_Minimum	0.012
wavelet_HLH_firstorder_Range	0.020
wavelet_HLH_glcm_Autocorrelation	0.013
wavelet_HLH_glcm_JointAverage	0.013
wavelet_HLH_glcm_Correlation	0.004
wavelet_HLH_glcm_Imc1	0.007
wavelet_HLH_glcm_Imc2	0.009
wavelet_HLH_glcm_Idm	0.036
wavelet_HLH_glcm_Id	0.039
wavelet_HLH_glcm_InverseVariance	0.046
wavelet_HLH_glcm_MaximumProbability	0.033
wavelet_HLH_glrlm_GrayLevelNonUniformity	0.002
wavelet_HLH_glrlm_HighGrayLevelRunEmphasis	0.014
wavelet_HLH_glrlm_LongRunHighGrayLevelEmphasis	0.031
wavelet_HLH_glrlm_ShortRunHighGrayLevelEmphasis	0.013
wavelet_HLH_glszm_HighGrayLevelZoneEmphasis	0.015
wavelet_HLH_glszm_SmallAreaHighGrayLevelEmphasis	0.017
wavelet_HLH_gldm_DependenceVariance	0.011
wavelet_HLH_gldm_GrayLevelNonUniformity	0.015
wavelet_HLH_gldm_HighGrayLevelEmphasis	0.014
wavelet_HLH_gldm_SmallDependenceHighGrayLevelEmphasis	0.030
wavelet_HHL_firstorder_InterquartileRange	0.044

wavelet_HHL_firstorder_Maximum	0.028
wavelet_HHL_glcm_Correlation	0.004
wavelet_HHL_glcm_Imc1	0.002
wavelet_HHL_glcm_Imc2	0.002
wavelet_HHL_glcm_Idm	0.037
wavelet_HHL_glcm_Id	0.039
wavelet_HHL_glcm_InverseVariance	0.040
wavelet_HHL_glcm_MaximumProbability	0.040
wavelet_HHL_glrlm_GrayLevelNonUniformity	0.003
wavelet_HHL_gldm_DependenceNonUniformityNormalized	0.041
wavelet_HHL_gldm_DependenceVariance	0.016
wavelet_HHL_gldm_GrayLevelNonUniformity	0.014
wavelet_HHL_gldm_SmallDependenceHighGrayLevelEmphasis	0.038
wavelet_HHH_firstorder_10Percentile	0.038
wavelet_HHH_firstorder_90Percentile	0.045
wavelet_HHH_firstorder_InterquartileRange	0.017
wavelet_HHH_firstorder_Kurtosis	0.003
wavelet_HHH_firstorder_Maximum	0.024
wavelet_HHH_firstorder_MeanAbsoluteDeviation	0.043
wavelet_HHH_firstorder_Minimum	0.026
wavelet_HHH_firstorder_Range	0.022
wavelet_HHH_firstorder_RobustMeanAbsoluteDeviation	0.022
wavelet_HHH_glcm_Autocorrelation	0.023
wavelet_HHH_glcm_JointAverage	0.027
wavelet_HHH_glcm_Correlation	0.000
wavelet_HHH_glcm_DifferenceAverage	0.029
wavelet_HHH_glcm_DifferenceEntropy	0.043
wavelet_HHH_glcm_JointEnergy	0.038
wavelet_HHH_glcm_JointEntropy	0.033
wavelet_HHH_glcm_Imc1	0.000
wavelet_HHH_glcm_Imc2	0.011
wavelet_HHH_glcm_Idm	0.019
wavelet_HHH_glcm_Id	0.019
wavelet_HHH_glcm_InverseVariance	0.032
wavelet_HHH_glcm_MaximumProbability	0.016
wavelet_HHH_glcm_SumEntropy	0.045
wavelet_HHH_glrlm_GrayLevelNonUniformity	0.001
wavelet_HHH_glrlm_HighGrayLevelRunEmphasis	0.023
wavelet_HHH_glrlm_RunLengthNonUniformityNormalized	0.045
wavelet_HHH_glrlm_RunPercentage	0.041
wavelet_HHH_glrlm_RunVariance	0.047
wavelet_HHH_glrlm_ShortRunHighGrayLevelEmphasis	0.022
wavelet_HHH_glszm_HighGrayLevelZoneEmphasis	0.024
wavelet_HHH_glszm_LargeAreaHighGrayLevelEmphasis	0.038

wavelet_HHH_glszm_SmallAreaEmphasis	0.000
wavelet_HHH_glszm_SmallAreaHighGrayLevelEmphasis	0.021
wavelet_HHH_gldm_DependenceNonUniformityNormalized	0.044
wavelet_HHH_gldm_DependenceVariance	0.002
wavelet_HHH_gldm_GrayLevelNonUniformity	0.004
wavelet_HHH_gldm_HighGrayLevelEmphasis	0.023
wavelet_HHH_gldm_LargeDependenceEmphasis	0.036
wavelet_LLL_firstorder_Energy	0.000
wavelet_LLL_firstorder_Median	0.042
wavelet_LLL_firstorder_Skewness	0.001
wavelet_LLL_firstorder_TotalEnergy	0.000
wavelet_LLL_glcm_Correlation	0.013
wavelet_LLL_glcm_DifferenceVariance	0.031
wavelet_LLL_glcm_JointEntropy	0.041
wavelet_LLL_glcm_Imc1	0.006
wavelet_LLL_glcm_Imc2	0.010
wavelet_LLL_glcm_Idmn	0.004
wavelet_LLL_glcm_Idn	0.027
wavelet_LLL_glrlm_GrayLevelNonUniformity	0.005
wavelet_LLL_gldm_GrayLevelNonUniformity	0.016

Supplementary Table 2. The univariate analysis of the relationship between radiomics features and Ki-67 index in surgically resected meningioma cases. The t test suggested that 349 of them were statistically different in high Ki-67 group and low Ki-67 group.

Radiomics Feature	p
original_shape_SurfaceVolumeRatio	0.024
original_firstorder_Median	0.038
original_firstorder_Skewness	0.000
original_glcm_Imc1	0.037
original_glcm_Idmn	0.024
original_glrlm_GrayLevelNonUniformity	0.002
original_glszm_SizeZoneNonUniformityNormalized	0.003
original_glszm_SmallAreaEmphasis	0.003
original_gldm_GrayLevelNonUniformity	0.015
original_gldm_LargeDependenceHighGrayLevelEmphasis	0.006
log_sigma_1_0_mm_3D_firstorder_Kurtosis	0.033
log_sigma_1_0_mm_3D_firstorder_Mean	0.034
log_sigma_1_0_mm_3D_firstorder_Median	0.041
log_sigma_1_0_mm_3D_firstorder_RootMeanSquared	0.027
log_sigma_1_0_mm_3D_glcm_Autocorrelation	0.042
log_sigma_1_0_mm_3D_glcm_JointAverage	0.030
log_sigma_1_0_mm_3D_glcm_Imc1	0.019
log_sigma_1_0_mm_3D_glcm_Imc2	0.009
log_sigma_1_0_mm_3D_glcm_Idmn	0.025
log_sigma_1_0_mm_3D_glcm_Idn	0.030
log_sigma_1_0_mm_3D_glrlm_GrayLevelNonUniformity	0.002
log_sigma_1_0_mm_3D_glrlm_LongRunHighGrayLevelEmphasis	0.044
log_sigma_1_0_mm_3D_glszm_SizeZoneNonUniformityNormalized	0.016
log_sigma_1_0_mm_3D_glszm_SmallAreaEmphasis	0.013
log_sigma_1_0_mm_3D_glszm_ZoneEntropy	0.030
log_sigma_1_0_mm_3D_gldm_GrayLevelNonUniformity	0.017
log_sigma_2_0_mm_3D_firstorder_10Percentile	0.017
log_sigma_2_0_mm_3D_firstorder_InterquartileRange	0.040
log_sigma_2_0_mm_3D_firstorder_Kurtosis	0.030
log_sigma_2_0_mm_3D_firstorder_Mean	0.002
log_sigma_2_0_mm_3D_firstorder_Median	0.001
log_sigma_2_0_mm_3D_firstorder_RootMeanSquared	0.002
log_sigma_2_0_mm_3D_firstorder_Skewness	0.010
log_sigma_2_0_mm_3D_glcm_Autocorrelation	0.003
log_sigma_2_0_mm_3D_glcm_JointAverage	0.003
log_sigma_2_0_mm_3D_glcm_Correlation	0.005
log_sigma_2_0_mm_3D_glcm_DifferenceAverage	0.018

log_sigma_2_0_mm_3D_glcm_DifferenceEntropy	0.026
log_sigma_2_0_mm_3D_glcm_Imc1	0.004
log_sigma_2_0_mm_3D_glcm_Imc2	0.021
log_sigma_2_0_mm_3D_glcm_Idm	0.015
log_sigma_2_0_mm_3D_glcm_Idmn	0.005
log_sigma_2_0_mm_3D_glcm_Id	0.015
log_sigma_2_0_mm_3D_glcm_Idn	0.001
log_sigma_2_0_mm_3D_glcm_InverseVariance	0.004
log_sigma_2_0_mm_3D_glrlm_GrayLevelNonUniformity	0.001
log_sigma_2_0_mm_3D_glrlm_HighGrayLevelRunEmphasis	0.005
log_sigma_2_0_mm_3D_glrlm_LongRunHighGrayLevelEmphasis	0.005
log_sigma_2_0_mm_3D_glrlm_RunLengthNonUniformityNormalized	0.012
log_sigma_2_0_mm_3D_glrlm_RunPercentage	0.035
log_sigma_2_0_mm_3D_glrlm_ShortRunEmphasis	0.019
log_sigma_2_0_mm_3D_glrlm_ShortRunHighGrayLevelEmphasis	0.009
log_sigma_2_0_mm_3D_glszm_HighGrayLevelZoneEmphasis	0.018
log_sigma_2_0_mm_3D_glszm_SizeZoneNonUniformity	0.001
log_sigma_2_0_mm_3D_glszm_SizeZoneNonUniformityNormalized	0.024
log_sigma_2_0_mm_3D_glszm_SmallAreaEmphasis	0.021
log_sigma_2_0_mm_3D_glszm_ZoneEntropy	0.033
log_sigma_2_0_mm_3D_glszm_ZonePercentage	0.006
log_sigma_2_0_mm_3D_gldm_DependenceEntropy	0.024
log_sigma_2_0_mm_3D_gldm_DependenceNonUniformityNormalized	0.010
log_sigma_2_0_mm_3D_gldm_GrayLevelNonUniformity	0.008
log_sigma_2_0_mm_3D_gldm_HighGrayLevelEmphasis	0.004
log_sigma_2_0_mm_3D_gldm_LargeDependenceHighGrayLevelEmphasis	0.005
log_sigma_2_0_mm_3D_gldm_SmallDependenceEmphasis	0.006
log_sigma_3_0_mm_3D_firstorder_10Percentile	0.004
log_sigma_3_0_mm_3D_firstorder_90Percentile	0.003
log_sigma_3_0_mm_3D_firstorder_Mean	0.000
log_sigma_3_0_mm_3D_firstorder_Median	0.000
log_sigma_3_0_mm_3D_firstorder_RootMeanSquared	0.000
log_sigma_3_0_mm_3D_firstorder_Skewness	0.019
log_sigma_3_0_mm_3D_glcm_Contrast	0.015
log_sigma_3_0_mm_3D_glcm_Correlation	0.000
log_sigma_3_0_mm_3D_glcm_DifferenceAverage	0.005
log_sigma_3_0_mm_3D_glcm_DifferenceEntropy	0.005
log_sigma_3_0_mm_3D_glcm_DifferenceVariance	0.018
log_sigma_3_0_mm_3D_glcm_Imc2	0.000
log_sigma_3_0_mm_3D_glcm_Idm	0.004
log_sigma_3_0_mm_3D_glcm_Idmn	0.018
log_sigma_3_0_mm_3D_glcm_Id	0.004
log_sigma_3_0_mm_3D_glcm_Idn	0.004
log_sigma_3_0_mm_3D_glcm_InverseVariance	0.002

log_sigma_3_0_mm_3D_glrlm_GrayLevelNonUniformity	0.000
log_sigma_3_0_mm_3D_glrlm_RunLengthNonUniformityNormalized	0.003
log_sigma_3_0_mm_3D_glrlm_RunPercentage	0.007
log_sigma_3_0_mm_3D_glrlm_ShortRunEmphasis	0.005
log_sigma_3_0_mm_3D_glszm_GrayLevelNonUniformity	0.000
log_sigma_3_0_mm_3D_glszm_SizeZoneNonUniformity	0.012
log_sigma_3_0_mm_3D_glszm_SizeZoneNonUniformityNormalized	0.028
log_sigma_3_0_mm_3D_glszm_SmallAreaEmphasis	0.031
log_sigma_3_0_mm_3D_glszm_ZonePercentage	0.003
log_sigma_3_0_mm_3D_gldm_DependenceEntropy	0.006
log_sigma_3_0_mm_3D_gldm_DependenceNonUniformityNormalized	0.007
log_sigma_3_0_mm_3D_gldm_DependenceVariance	0.037
log_sigma_3_0_mm_3D_gldm_GrayLevelNonUniformity	0.005
log_sigma_3_0_mm_3D_gldm_LargeDependenceEmphasis	0.026
log_sigma_3_0_mm_3D_gldm_LargeDependenceHighGrayLevelEmphasis	0.027
log_sigma_3_0_mm_3D_gldm_SmallDependenceEmphasis	0.003
log_sigma_4_0_mm_3D_firstorder_10Percentile	0.001
log_sigma_4_0_mm_3D_firstorder_90Percentile	0.001
log_sigma_4_0_mm_3D_firstorder_InterquartileRange	0.020
log_sigma_4_0_mm_3D_firstorder_MeanAbsoluteDeviation	0.046
log_sigma_4_0_mm_3D_firstorder_Mean	0.000
log_sigma_4_0_mm_3D_firstorder_RobustMeanAbsoluteDeviation	0.026
log_sigma_4_0_mm_3D_glcm_Contrast	0.004
log_sigma_4_0_mm_3D_glcm_Correlation	0.001
log_sigma_4_0_mm_3D_glcm_DifferenceAverage	0.002
log_sigma_4_0_mm_3D_glcm_DifferenceEntropy	0.001
log_sigma_4_0_mm_3D_glcm_DifferenceVariance	0.002
log_sigma_4_0_mm_3D_glcm_JointEntropy	0.034
log_sigma_4_0_mm_3D_glcm_Imc2	0.001
log_sigma_4_0_mm_3D_glcm_Idm	0.002
log_sigma_4_0_mm_3D_glcm_Idmn	0.010
log_sigma_4_0_mm_3D_glcm_Id	0.002
log_sigma_4_0_mm_3D_glcm_Idn	0.002
log_sigma_4_0_mm_3D_glcm_InverseVariance	0.001
log_sigma_4_0_mm_3D_glrlm_GrayLevelNonUniformity	0.000
log_sigma_4_0_mm_3D_glrlm_LongRunEmphasis	0.035
log_sigma_4_0_mm_3D_glrlm_RunLengthNonUniformityNormalized	0.002
log_sigma_4_0_mm_3D_glrlm_RunPercentage	0.003
log_sigma_4_0_mm_3D_glrlm_ShortRunEmphasis	0.003
log_sigma_4_0_mm_3D_glszm_GrayLevelNonUniformity	0.003
log_sigma_4_0_mm_3D_glszm_SizeZoneNonUniformityNormalized	0.018
log_sigma_4_0_mm_3D_glszm_SmallAreaEmphasis	0.027
log_sigma_4_0_mm_3D_glszm_ZonePercentage	0.001
log_sigma_4_0_mm_3D_gldm_DependenceEntropy	0.017

log_sigma_4_0_mm_3D_gldm_DependenceNonUniformityNormalized	0.008
log_sigma_4_0_mm_3D_gldm_DependenceVariance	0.023
log_sigma_4_0_mm_3D_gldm_GrayLevelNonUniformity	0.003
log_sigma_4_0_mm_3D_gldm_LargeDependenceEmphasis	0.013
log_sigma_4_0_mm_3D_gldm_LargeDependenceHighGrayLevelEmphasis	0.040
log_sigma_4_0_mm_3D_gldm_SmallDependenceEmphasis	0.001
log_sigma_5_0_mm_3D_firstorder_10Percentile	0.000
log_sigma_5_0_mm_3D_firstorder_90Percentile	0.002
log_sigma_5_0_mm_3D_firstorder_Entropy	0.014
log_sigma_5_0_mm_3D_firstorder_InterquartileRange	0.001
log_sigma_5_0_mm_3D_firstorder_Kurtosis	0.011
log_sigma_5_0_mm_3D_firstorder_MeanAbsoluteDeviation	0.004
log_sigma_5_0_mm_3D_firstorder_Minimum	0.044
log_sigma_5_0_mm_3D_firstorder_RobustMeanAbsoluteDeviation	0.001
log_sigma_5_0_mm_3D_firstorder_Uniformity	0.013
log_sigma_5_0_mm_3D_firstorder_Variance	0.026
log_sigma_5_0_mm_3D_glcm_Contrast	0.001
log_sigma_5_0_mm_3D_glcm_Correlation	0.026
log_sigma_5_0_mm_3D_glcm_DifferenceAverage	0.000
log_sigma_5_0_mm_3D_glcm_DifferenceEntropy	0.000
log_sigma_5_0_mm_3D_glcm_DifferenceVariance	0.001
log_sigma_5_0_mm_3D_glcm_JointEnergy	0.029
log_sigma_5_0_mm_3D_glcm_JointEntropy	0.006
log_sigma_5_0_mm_3D_glcm_Imc1	0.000
log_sigma_5_0_mm_3D_glcm_Idm	0.000
log_sigma_5_0_mm_3D_glcm_Idmn	0.005
log_sigma_5_0_mm_3D_glcm_Id	0.000
log_sigma_5_0_mm_3D_glcm_Idn	0.001
log_sigma_5_0_mm_3D_glcm_InverseVariance	0.000
log_sigma_5_0_mm_3D_glcm_SumEntropy	0.033
log_sigma_5_0_mm_3D_glrlm_GrayLevelNonUniformity	0.000
log_sigma_5_0_mm_3D_glrlm_GrayLevelNonUniformityNormalized	0.016
log_sigma_5_0_mm_3D_glrlm_GrayLevelVariance	0.028
log_sigma_5_0_mm_3D_glrlm_LongRunEmphasis	0.013
log_sigma_5_0_mm_3D_glrlm_RunLengthNonUniformityNormalized	0.000
log_sigma_5_0_mm_3D_glrlm_RunPercentage	0.001
log_sigma_5_0_mm_3D_glrlm_RunVariance	0.019
log_sigma_5_0_mm_3D_glrlm_ShortRunEmphasis	0.001
log_sigma_5_0_mm_3D_glszm_GrayLevelNonUniformity	0.023
log_sigma_5_0_mm_3D_glszm_ZonePercentage	0.000
log_sigma_5_0_mm_3D_gldm_DependenceEntropy	0.018
log_sigma_5_0_mm_3D_gldm_DependenceNonUniformityNormalized	0.000
log_sigma_5_0_mm_3D_gldm_DependenceVariance	0.006
log_sigma_5_0_mm_3D_gldm_GrayLevelNonUniformity	0.002

log_sigma_5_0_mm_3D_gldm_GrayLevelVariance	0.026
log_sigma_5_0_mm_3D_gldm_LargeDependenceEmphasis	0.004
log_sigma_5_0_mm_3D_gldm_LargeDependenceHighGrayLevelEmphasis	0.011
log_sigma_5_0_mm_3D_gldm_SmallDependenceEmphasis	0.000
log_sigma_5_0_mm_3D_gldm_SmallDependenceHighGrayLevelEmphasis	0.024
log_sigma_5_0_mm_3D_gldm_SmallDependenceLowGrayLevelEmphasis	0.049
wavelet_LLH_firstorder_90Percentile	0.047
wavelet_LLH_firstorder_Maximum	0.000
wavelet_LLH_firstorder_Mean	0.007
wavelet_LLH_firstorder_Minimum	0.029
wavelet_LLH_firstorder_Range	0.003
wavelet_LLH_firstorder_RootMeanSquared	0.000
wavelet_LLH_glcm_Autocorrelation	0.029
wavelet_LLH_glcm_JointAverage	0.017
wavelet_LLH_glcm_Correlation	0.001
wavelet_LLH_glcm_Imc1	0.040
wavelet_LLH_glcm_Imc2	0.004
wavelet_LLH_glrlm_GrayLevelNonUniformity	0.006
wavelet_LLH_glrlm_HighGrayLevelRunEmphasis	0.029
wavelet_LLH_glrlm_LongRunHighGrayLevelEmphasis	0.035
wavelet_LLH_glrlm_ShortRunHighGrayLevelEmphasis	0.029
wavelet_LLH_glszm_HighGrayLevelZoneEmphasis	0.024
wavelet_LLH_glszm_SmallAreaHighGrayLevelEmphasis	0.038
wavelet_LLH_gldm_GrayLevelNonUniformity	0.025
wavelet_LLH_gldm_HighGrayLevelEmphasis	0.029
wavelet_LHL_firstorder_Maximum	0.034
wavelet_LHL_firstorder_Minimum	0.043
wavelet_LHL_firstorder_Range	0.032
wavelet_LHL_firstorder_RootMeanSquared	0.014
wavelet_LHL_glcm_Autocorrelation	0.029
wavelet_LHL_glcm_JointAverage	0.033
wavelet_LHL_glcm_Correlation	0.001
wavelet_LHL_glcm_Imc1	0.006
wavelet_LHL_glcm_Imc2	0.000
wavelet_LHL_glrlm_GrayLevelNonUniformity	0.005
wavelet_LHL_glrlm_HighGrayLevelRunEmphasis	0.030
wavelet_LHL_glrlm_LongRunHighGrayLevelEmphasis	0.044
wavelet_LHL_glrlm_ShortRunHighGrayLevelEmphasis	0.029
wavelet_LHL_glszm_HighGrayLevelZoneEmphasis	0.028
wavelet_LHL_glszm_SmallAreaHighGrayLevelEmphasis	0.038
wavelet_LHL_gldm_GrayLevelNonUniformity	0.015
wavelet_LHL_gldm_HighGrayLevelEmphasis	0.030
wavelet_LHL_gldm_SmallDependenceHighGrayLevelEmphasis	0.042
wavelet_LHH_firstorder_10Percentile	0.039

wavelet_LHH_firstorder_InterquartileRange	0.025
wavelet_LHH_firstorder_Maximum	0.016
wavelet_LHH_firstorder_Minimum	0.012
wavelet_LHH_firstorder_Range	0.012
wavelet_LHH_firstorder_RobustMeanAbsoluteDeviation	0.031
wavelet_LHH_glcm_Autocorrelation	0.013
wavelet_LHH_glcm_JointAverage	0.010
wavelet_LHH_glcm_Contrast	0.042
wavelet_LHH_glcm_Correlation	0.000
wavelet_LHH_glcm_DifferenceAverage	0.030
wavelet_LHH_glcm_JointEntropy	0.047
wavelet_LHH_glcm_Imc1	0.002
wavelet_LHH_glcm_Imc2	0.001
wavelet_LHH_glcm_Idm	0.021
wavelet_LHH_glcm_Id	0.022
wavelet_LHH_glcm_InverseVariance	0.021
wavelet_LHH_glcm_MaximumProbability	0.030
wavelet_LHH_glrlm_GrayLevelNonUniformity	0.002
wavelet_LHH_glrlm_HighGrayLevelRunEmphasis	0.013
wavelet_LHH_glrlm_LongRunHighGrayLevelEmphasis	0.031
wavelet_LHH_glrlm_ShortRunHighGrayLevelEmphasis	0.012
wavelet_LHH_glszm_HighGrayLevelZoneEmphasis	0.014
wavelet_LHH_glszm_SmallAreaHighGrayLevelEmphasis	0.017
wavelet_LHH_gldm_DependenceNonUniformityNormalized	0.027
wavelet_LHH_gldm_DependenceVariance	0.009
wavelet_LHH_gldm_GrayLevelNonUniformity	0.010
wavelet_LHH_gldm_HighGrayLevelEmphasis	0.013
wavelet_LHH_gldm_LargeDependenceEmphasis	0.047
wavelet_LHH_gldm_SmallDependenceHighGrayLevelEmphasis	0.026
wavelet_HLL_firstorder_Maximum	0.033
wavelet_HLL_firstorder_Mean	0.002
wavelet_HLL_firstorder_Median	0.022
wavelet_HLL_firstorder_Minimum	0.047
wavelet_HLL_firstorder_Range	0.031
wavelet_HLL_firstorder_RootMeanSquared	0.000
wavelet_HLL_glcm_Autocorrelation	0.023
wavelet_HLL_glcm_JointAverage	0.034
wavelet_HLL_glcm_Correlation	0.016
wavelet_HLL_glcm_Imc1	0.008
wavelet_HLL_glcm_Imc2	0.001
wavelet_HLL_glrlm_GrayLevelNonUniformity	0.009
wavelet_HLL_glrlm_HighGrayLevelRunEmphasis	0.024
wavelet_HLL_glrlm_LongRunHighGrayLevelEmphasis	0.038
wavelet_HLL_glrlm_ShortRunHighGrayLevelEmphasis	0.023

wavelet_HLL_glszm_HighGrayLevelZoneEmphasis	0.023
wavelet_HLL_glszm_SmallAreaHighGrayLevelEmphasis	0.028
wavelet_HLL_gldm_GrayLevelNonUniformity	0.029
wavelet_HLL_gldm_HighGrayLevelEmphasis	0.024
wavelet_HLL_gldm_SmallDependenceHighGrayLevelEmphasis	0.032
wavelet_HLH_firstorder_Maximum	0.045
wavelet_HLH_firstorder_Minimum	0.012
wavelet_HLH_firstorder_Range	0.020
wavelet_HLH_glcm_Autocorrelation	0.013
wavelet_HLH_glcm_JointAverage	0.013
wavelet_HLH_glcm_Correlation	0.004
wavelet_HLH_glcm_Imc1	0.007
wavelet_HLH_glcm_Imc2	0.009
wavelet_HLH_glcm_Idm	0.036
wavelet_HLH_glcm_Id	0.039
wavelet_HLH_glcm_InverseVariance	0.046
wavelet_HLH_glcm_MaximumProbability	0.033
wavelet_HLH_glrlm_GrayLevelNonUniformity	0.002
wavelet_HLH_glrlm_HighGrayLevelRunEmphasis	0.014
wavelet_HLH_glrlm_LongRunHighGrayLevelEmphasis	0.031
wavelet_HLH_glrlm_ShortRunHighGrayLevelEmphasis	0.013
wavelet_HLH_glszm_HighGrayLevelZoneEmphasis	0.015
wavelet_HLH_glszm_SmallAreaHighGrayLevelEmphasis	0.017
wavelet_HLH_gldm_DependenceVariance	0.011
wavelet_HLH_gldm_GrayLevelNonUniformity	0.015
wavelet_HLH_gldm_HighGrayLevelEmphasis	0.014
wavelet_HLH_gldm_SmallDependenceHighGrayLevelEmphasis	0.030
wavelet_HHL_firstorder_InterquartileRange	0.044
wavelet_HHL_firstorder_Maximum	0.028
wavelet_HHL_glcm_Correlation	0.004
wavelet_HHL_glcm_Imc1	0.002
wavelet_HHL_glcm_Imc2	0.002
wavelet_HHL_glcm_Idm	0.037
wavelet_HHL_glcm_Id	0.039
wavelet_HHL_glcm_InverseVariance	0.040
wavelet_HHL_glcm_MaximumProbability	0.040
wavelet_HHL_glrlm_GrayLevelNonUniformity	0.003
wavelet_HHL_gldm_DependenceNonUniformityNormalized	0.041
wavelet_HHL_gldm_DependenceVariance	0.016
wavelet_HHL_gldm_GrayLevelNonUniformity	0.014
wavelet_HHL_gldm_SmallDependenceHighGrayLevelEmphasis	0.038
wavelet_HHH_firstorder_10Percentile	0.038
wavelet_HHH_firstorder_90Percentile	0.045
wavelet_HHH_firstorder_InterquartileRange	0.017

wavelet_HHH_firstorder_Kurtosis	0.003
wavelet_HHH_firstorder_Maximum	0.024
wavelet_HHH_firstorder_MeanAbsoluteDeviation	0.043
wavelet_HHH_firstorder_Minimum	0.026
wavelet_HHH_firstorder_Range	0.022
wavelet_HHH_firstorder_RobustMeanAbsoluteDeviation	0.022
wavelet_HHH_glcm_Autocorrelation	0.023
wavelet_HHH_glcm_JointAverage	0.027
wavelet_HHH_glcm_Correlation	0.000
wavelet_HHH_glcm_DifferenceAverage	0.029
wavelet_HHH_glcm_DifferenceEntropy	0.043
wavelet_HHH_glcm_JointEnergy	0.038
wavelet_HHH_glcm_JointEntropy	0.033
wavelet_HHH_glcm_Imc1	0.000
wavelet_HHH_glcm_Imc2	0.011
wavelet_HHH_glcm_Idm	0.019
wavelet_HHH_glcm_Id	0.019
wavelet_HHH_glcm_InverseVariance	0.032
wavelet_HHH_glcm_MaximumProbability	0.016
wavelet_HHH_glcm_SumEntropy	0.045
wavelet_HHH_glrlm_GrayLevelNonUniformity	0.001
wavelet_HHH_glrlm_HighGrayLevelRunEmphasis	0.023
wavelet_HHH_glrlm_RunLengthNonUniformityNormalized	0.045
wavelet_HHH_glrlm_RunPercentage	0.041
wavelet_HHH_glrlm_RunVariance	0.047
wavelet_HHH_glrlm_ShortRunHighGrayLevelEmphasis	0.022
wavelet_HHH_glszm_HighGrayLevelZoneEmphasis	0.024
wavelet_HHH_glszm_LargeAreaHighGrayLevelEmphasis	0.038
wavelet_HHH_glszm_SmallAreaEmphasis	0.000
wavelet_HHH_glszm_SmallAreaHighGrayLevelEmphasis	0.021
wavelet_HHH_gldm_DependenceNonUniformityNormalized	0.044
wavelet_HHH_gldm_DependenceVariance	0.002
wavelet_HHH_gldm_GrayLevelNonUniformity	0.004
wavelet_HHH_gldm_HighGrayLevelEmphasis	0.023
wavelet_HHH_gldm_LargeDependenceEmphasis	0.036
wavelet_LLL_firstorder_Energy	0.000
wavelet_LLL_firstorder_Median	0.042
wavelet_LLL_firstorder_Skewness	0.001
wavelet_LLL_firstorder_TotalEnergy	0.000
wavelet_LLL_glcm_Correlation	0.013
wavelet_LLL_glcm_DifferenceVariance	0.031
wavelet_LLL_glcm_JointEntropy	0.041
wavelet_LLL_glcm_Imc1	0.006
wavelet_LLL_glcm_Imc2	0.010

wavelet_LLL_glcm_Idmn	0.004
wavelet_LLL_glcm_Idn	0.027
wavelet_LLL_glrlm_GrayLevelNonUniformity	0.005
wavelet_LLL_gldm_GrayLevelNonUniformity	0.016

GLCM: Gray-level co-occurrence matrix; GLRLM: Gray-level run length matrix; GLSZM: Gray-level size zone matrix; GLDM: Gray-level dependence matrix.

Supplementary Table 3. The univariate analysis of the relationship between radiological characteristics and Ki-67 index in surgically resected meningioma cases.

	< 5% (low group)	≥ 5% (high group)	p values
**Number**	556	167	
**Tumor volume (cm^3)**	27.0 ± 25.9 (IQR: 9.2-35.3)	46.3 ± 48.2 (IQR: 13.7-57.8)	<0.001
**Peritumoral edema**			
EI=1	296 (53.2%)	76 (45.5%)	<0.001
1<EI<2	144 (25.9%)	28 (16.8%)	
2≤EI< 3	72 (12.9%)	28 (16.8%)	
EI≥3	44 (7.9%)	35 (20.9%)	
**CSF cleft surrounding tumor**	387 (69.6%)	109 (65.3%)	0.493
**Absent capsular enhancement**	131 (23.6%)	48 (28.7%)	0.315
**Intra-tumoral necrosis**	104 (18.7%)	69 (41.3%)	0.008

EI = Edema Index; CSF = cerebral spinal fluid

Supplementary Table 4. The multivariate analysis of traditional radiological characteristics by using logistic regression. The tumor volume was stratified based on the optimal cutoff values of 32.9, determined at the point of the maximal Youden index calculated by receiver operating characteristic (ROC) analyses.

Variables	Odds ratio (95CI)	coefficient	P value
Tumor volume	1.684 (0.999-2.839)	0.521	0.091
Peritumoral edema	1.229 (0.995-1.519)	0.206	0.156
Intra-tumoral necrosis	4.048 (2.878-5.694)	1.164	<0.001

Supplementary Table 5. Comparison between the multi-modal learning representing model with existing AI methods for predicting Ki-67 of meningiomas in internal performance.

Input	Modeling	AUC (95CI)	Accuracy (95CI)	Sensitivity (95CI)	Specificity (95CI)	F1 score (95CI)	MCC (95CI)
Radiological characteristics	Logistic Regression	0.664 (0.624-0.710)	0.614 (0.582-0.651)	0.602 (0.563-0.648)	0.629 (0.563-0.648)	0.607 (0.573-0.642)	0.224 (0.157-0.292)
MRI	ResNet 50	0.690 (0.656-0.727)	0.600 (0.567-0.637)	0.819 (0.779-0.863)	0.306 (0.267-0.349)	0.686 (0.651-0.720)	0.187 (0.116-0.250)
Radiomics	Lasso + SVM	0.708 (0.665-0.751)	0.655 (0.615-0.699)	0.783 (0.731-0.839)	0.484 (0.429-0.544)	0.720 (0.682-0.763)	0.258 (0.191-0.333)
Radiological characteristic /radiomics	Lasso + SVM	0.733 (0.696-0.770)	0.676 (0.643-0.713)	0.795 (0.745-0.842)	0.516 (0.457-0.572)	0.728 (0.494-0.563)	0.418 (0.302-0.439)
Radiomics	Lasso + LDA	0.724 (0.676-0.767)	0.669 (0.622-0.713)	0.687 (0.625-0.746)	0.645 (0.584-0.717)	0.678 (0.637-0.720)	0.292 (0.221-0.368)
Radiological characteristic /radiomics	Lasso + LDA	0.746 (0.717-0.780)	0.655 (0.629-0.685)	0.675 (0.635-0.719)	0.629 (0.590-0.672)	0.675 (0.642-0.705)	0.299 (0.236-0.361)

Supplementary Table 6. Comparison between the multi-modal learning representing model with existing AI methods for predicting Ki-67 of meningiomas in external generalization.

Input	Modeling	AUC (95CI)	Accuracy (95CI)	Sensitivity (95CI)	Specificity (95CI)	F1 (95CI)	MCC (95CI)
Radiological characteristics	Logistic Regression	0.506 (0.439-0.587)	0.491 (0.356-0.627)	0.505 (0.356-0.713)	0.489 (0.387-0.513)	0.498 (0.430-0.580)	0.009 (-0.104-0.117)
MRI	ResNet 50	0.464 (0.363-0.565)	0.421 (0.354-0.477)	0.750 (0.619-0.869)	0.367 (0.310-0.433)	0.545 (0.424-0.567)	0.066 (-0.101-0.126)
Radiomics	Lasso + SVM	0.538 (0.442-0.634)	0.540 (0.492-0.596)	0.542 (0.411-0.661)	0.536 (0.479-0.602)	0.540 (0.337-0.713)	0.178 (-0.027-0.165)
Radiological characteristic /radiomics	Lasso + SVM	0.711 (0.612-0.82)	0.710 (0.634-0.774)	0.750 (0.622-0.872)	0.676 (0.608-0.734)	0.742 (0.675-0.825)	0.404 (0.301-0.513)
Radiomics	Lasso + LDA	0.619 (0.526-0.718)	0.619 (0.560-0.683)	0.634 (0.506-0.756)	0.606 (0.559-0.661)	0.625 (0.477-0.784)	0.266 (0.137-0.398)
Radiological characteristic /radiomics	Lasso + LDA	0.676 (0.585-0.769)	0.817 (0.767-0.873)	0.314 (0.183-0.433)	0.915 (0.858-0.96)	0.423 (0.312-0.565)	0.351 (0.207-0.482)

Supplementary Table 7. Univariate and multivariable Cox regression analysis of predictors of tumor volume growth in 5 years.

Variables	Univariate analysis		Multivariate analysis (Output 1 with others)		Multivariate analysis (Output 2 with others)	
	HR (95% CI)	p - value	HR (95% CI)	p - value	HR (95% CI)	p - value
**Output 1 Prediction**	3.043 (1.818-5.096)	<0.001	1.996 (1.117-3.568)	0.019	-	-
**Output 2 Prediction**	2.782 (1.647-4.703)	0.004	-	-	2.539 (1.470-4.387)	<0.001
**Absent CSF cleft**	1.660 (0.841-3.278)	0.145	1.655 (0.831-3.332)	0.152	1.889 (0.944-3.779)	0.072
**Absent capsular enhancement**	0.948 (0.408-2.206)	0.902	1.246 (0.524-2.961)	0.619	1.244 (0.522-2.945)	0.627
**Intratumor necrosis**	6.814 (3.197-14.520)	0.013	1.256 (0.484-3.255)	0.640	1.342 (0.546-3.293)	0.523
**Peritumoral edema**	8.408 (5.536-12.772)	<0.001	7.522 (4.851-11.663)	<0.001	8.302 (5.277-13.059)	<0.001

Supplementary Table 8. Summary of the existing Ki-67 prediction research by using medical image analysis technology.

Authors	Years	Multi-center or single-center	Retrospective or perspective	Methodology	Model performance				
						AUC	ACC	Sensitivity	Specificity
Jiawei Chen, et al.	2023	multi-center	retrospective	fine-tuned ResNet50 (CNN)	Internal	0.966	0.916	0.959	0.959
					External	0.591	0.598	0.636	0.606
Chung-Man Moon, et al.	2023	multi-center	retrospective	Radiomics + machine learning	Internal	0.820	0.867	0.671	0.969
					External	0.712	0.743	0.643	0.780
Yanjie Zhao, et al.	2022	multi-center	retrospective	Radiomics + machine learning	Internal	0.837	0.810	0.857	0.771
					External	0.700	0.557	0.314	0.885
Omaditya Khanna, et al.	2021	single-center	retrospective	Radiomics + machine learning	Internal	0.840	-	0.841	0.733
